# Defective Notch1 signaling in endothelial cells drives pathogenesis in a mouse model of Adams-Oliver syndrome

**DOI:** 10.1172/JCI187532

**Published:** 2025-10-07

**Authors:** Alyssa F. Solano, Kristina Preusse, Brittany Cain, Rebecca Hotz, Parthav Gavini, Zhenyu Yuan, Benjamin Bowen, Gabrielle Maco, Hope Neal, Ellen K. Gagliani, Christopher Ahn, Hee-Woong Lim, Laura Southgate, Rhett A. Kovall, Raphael Kopan, Brian Gebelein

**Affiliations:** 1 Division of Developmental Biology, Cincinnati Children’s Hospital Medical Center, Cincinnati, Ohio, USA.; 2 Medical Scientist Training Program, University of Cincinnati College of Medicine, Cincinnati, Ohio, USA.; 3 Graduate Program in Molecular and Developmental Biology, Cincinnati Children’s Hospital Research Foundation, Cincinnati, Ohio, USA.; 4 Department of Molecular and Cellular Biosciences, University of Cincinnati College of Medicine, Cincinnati, Ohio, USA.; 5 Department of Chemistry, Xavier University, Cincinnati, Ohio, USA.; 6 Division of Biomedical Informatics, Cincinnati Children’s Hospital Medical Center, Cincinnati, Ohio, USA.; 7 Department of Pediatrics, University of Cincinnati College of Medicine, Cincinnati, Ohio, USA.; 8 School of Health and Medical Sciences, City St George’s, University of London, United Kingdom.; 9 Department of Medical and Molecular Genetics, Faculty of Life Sciences and Medicine, King’s College London, London, United Kingdom.

**Keywords:** Development, Vascular biology, Embryonic development, Genetic diseases, Mouse models

## Abstract

Adams-Oliver syndrome (AOS) is a rare congenital disorder characterized by scalp, limb, and cardiovascular defects. Although variants in the NOTCH1 receptor, DLL4 ligand, and RBPJ transcription factor have been implicated in AOS, the driving tissue types and molecular mechanisms by which these variants cause pathogenesis are unknown. Here, we used quantitative binding assays to show that AOS-associated RBPJ missense variants compromise DNA binding but not cofactor binding. These findings suggest that AOS-associated RBPJ variants do not function as loss-of-function alleles but instead act as dominant-negative proteins that sequester cofactors from DNA. Consistent with this idea, mice carrying an AOS-associated *Rbpj* allele develop dominant phenotypes that include increased lethality and cardiovascular defects in a *Notch1* heterozygous background, whereas *Notch1* and *Rbpj* compound heterozygous null alleles are well tolerated. To facilitate studies into the tissues driving AOS pathogenesis, we employed conditional genetics to isolate the contribution of the vascular endothelium to the development of AOS-like phenotypes. Importantly, our studies show that expression of the *Rbpj* AOS allele in endothelial cells is both necessary and sufficient to cause lethality and cardiovascular defects. These data establish that reduced Notch1 signaling in the vasculature is a key driver of pathogenesis in this AOS mouse model.

## Abstract 2

We show that RBPJ variants associated with Adams-Oliver Syndrome act as dominant-negative proteins that reduce Notch1 signaling in the endothelium to cause pathogenesis.

## Graphical abstract

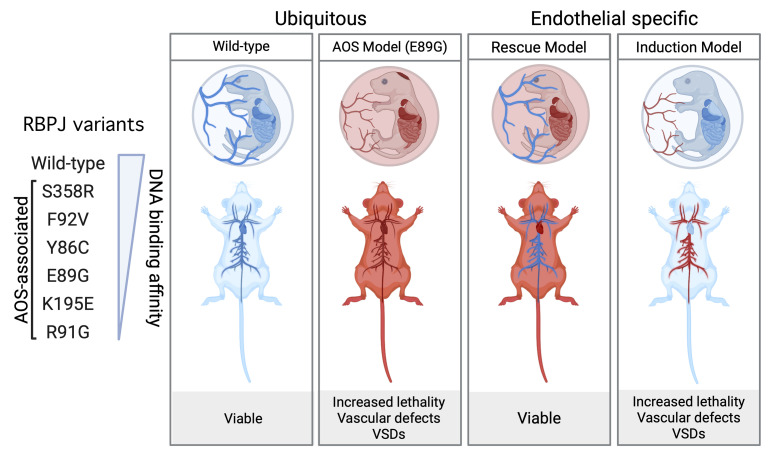

## Introduction

Adams-Oliver syndrome (AOS) is a rare congenital condition characterized by aplasia cutis congenita, which is a thinning and/or absence of skin and skull tissue at the top of the head, and transverse terminal limb truncations (1, 2). In addition, patients with AOS frequently present with heart and vascular defects such as atrial and ventricular septal defects, valve anomalies, aortic and pulmonic stenosis, coarctation of the aorta, patent ductus arteriosus, persistent truncus arteriosus, tetralogy of Fallot, cutis marmorata telangiectatica congenita, portal vein agenesis, portal hypertension, esophageal varices, intracranial hemorrhages, and thrombosis (2, 3). A smaller number of patients with AOS have neurological defects such as microcephaly, ventricular dilation, corpus callosum hypoplasia, periventricular lesions, visual deficits, epilepsy, spasticity, and cognitive impairment (2). Approximately 10% have intrauterine growth restriction (2). Hence, AOS features include a complex mixture of symptoms requiring a multidisciplinary approach to clinical management.

Genetic studies have revealed that approximately 40% of patients with AOS inherit variant alleles in 1 of 6 genes: *NOTCH1*, *DLL4*, *RBPJ*, *EOGT*, *DOCK6*, and *ARHGAP31* (2). AOS cases caused by variants in *NOTCH1*, *DLL4*, *RBPJ*, and *ARHGAP31* are autosomal dominant (4–7), whereas *EOGT* and *DOCK6* variants are autosomal recessive (8, 9). Of these genes, 4 encode components of the Notch signaling pathway, including the receptor NOTCH1, the ligand DLL4, the transcription factor RBPJ, and the EGF domain–specific O-linked N-acetylglucosamine transferase EOGT, which posttranslationally modifies Notch proteins (10). The remaining 2 genes encode proteins that regulate small GTPases, with *DOCK6* encoding a guanine nucleotide exchange factor and *ARHGAP31* encoding a Rho GTPase-activating protein (4, 8). The relationship between the Notch pathway and small GTPase regulators in AOS pathogenesis is unclear. However, patients with Notch pathway variants have a higher prevalence of cardiovascular defects (49% vs. 13%), whereas patients with pathogenic *DOCK6* variants have a higher prevalence of brain anomalies (91% vs. 19%) (2). Overall, AOS pathogenesis remains poorly understood, and no disease-modifying therapies are available.

The canonical Notch pathway converts ligand/receptor interactions into changes in gene expression. Signaling is initiated when a ligand (DLL1, DLL3, DLL4, JAG1, or JAG2 in mammals) on a signal-sending cell binds a receptor (NOTCH1, NOTCH2, NOTCH3, or NOTCH4 in mammals) on a signal-receiving cell (10). Force generated during ligand endocytosis induces a receptor conformation change that allows proteolytic cleavage within the NOTCH transmembrane region to release the Notch intracellular domain (NICD) into the cytoplasm (11). NICD then transits to the nucleus, forms a ternary complex with RBPJ and the coactivator MAML, and activates target genes (11, 12). Conversely, RBPJ can also directly bind corepressors that limit Notch target gene transcription (13–16). Thus, Notch signal strength is largely determined by the number of NICD molecules and competing corepressors within a cell (17–19).

Notch signaling is iteratively used throughout development to regulate the morphogenesis of many organs, including the heart (20), vasculature (21), hematopoietic system (22), nervous system (23), and somite-derived organs (24). In fact, clinical studies have implicated aberrant Notch signaling in an array of health disorders that include AOS, aortic valve disease, hypoplastic left heart syndrome, Alagille syndrome, cerebral autosomal dominant arteriopathy with subcortical infarcts and leukoencephalopathy (CADASIL), Hajdu-Cheney syndrome, spondylocostal dysostosis, and cancer (25, 26). How specific defects in the Notch pathway cause this array of disease is an active area of research.

Given the implication of Notch pathway genes in AOS and the observed vascular changes in patients with AOS (2, 27), some have speculated that impaired vascular development drives AOS pathogenesis (6, 28, 29). However, a vascular etiology for AOS has yet to be established, and the heart, skin/scalp, and limb defects found in AOS could be caused by defective Notch signaling in multiple cell types (25, 26). Unfortunately, loss of a *Notch1* allele in mice is not sufficient to recapitulate AOS-like phenotypes, whereas loss of a *Dll4* allele is so severe that heterozygotes rarely survive to birth due to catastrophic vascular defects (30, 31). Tissue-specific induction of *Dll4* heterozygosity within the second heart field has been used to bypass early lethality and model the impact of *Dll4* heterozygosity on mouse heart development (32), but the requirement for tissue specificity limits the applications of this model. Thus, we currently lack a good mouse model of AOS to study pathogenesis.

Molecular genetic studies of patients with AOS have revealed frameshift and early truncation defects in *NOTCH1* and *DLL4* likely to render each allele null (2). These findings are consistent with dominant *NOTCH1* and *DLL4* variants creating loss-of-function alleles and haploinsufficiency causing AOS (33). In contrast, all AOS-associated *RBPJ* variants are missense substitutions; no frameshift or nonsense *RBPJ* variants have been identified that would encode obvious null alleles. To understand the mechanisms by which AOS-associated *RBPJ* variants affect Notch signaling, we previously leveraged a *Drosophila* line with an E137V mutation in *Suppressor of Hairless* [*Su(H)*, fly ortholog of *RBPJ*] that is analogous to an AOS-associated variant in human *RBPJ* at residue E63 (5, 34). Intriguingly, a single *Su(H)*^E137V^ allele was sufficient to induce wing nicking, a phenotype not seen in flies with a single *Su(H)*-null allele. Moreover, the *Su(H)*^E137V^ allele dramatically enhanced a loss of sensory bristle phenotype associated with haploinsufficiency of the antagonistic *Hairless (H)* corepressor, whereas the *Su(H)*-null allele suppressed this phenotype (34, 35). Molecularly, we found that both the fly Su(H)^E137V^ protein and a mouse Rbpj^E89G^ protein that is analogous to the human RBPJ^E63G^ AOS variant decreased DNA binding but not NICD nor corepressor binding (34). Consistent with these findings, Rbpj^E89G^ did not activate Notch reporter expression as well as WT Rbpj, even though Rbpj^E89G^ is properly localized to the nucleus and interacts with full-length NICD1 and the Sharp corepressor as well as WT Rbpj in co-IP assays (34). Taken together, these *Drosophila*, cell culture, and biochemical findings suggest that *RBPJ* AOS alleles encode dominant-negative proteins that dysregulate Notch signaling by sequestering NICD and other cofactors from DNA. However, whether cofactor sequestration is consistent across all AOS-associated *RBPJ* variants and how this mechanism leads to the complex array of AOS symptoms in humans is not understood.

Here, we used quantitative DNA binding assays to show that all 6 AOS-associated *RBPJ* alleles encode proteins with defective DNA binding activity but with differing degrees of severity, ranging from a 3-fold decrease to complete loss in DNA binding. To assess how such alleles affect mammalian development, we made 2 mouse models that encoded analogous AOS-associated RBPJ variants with approximately 3-fold (RBPJ^S358R^) and approximately 6-fold (RBPJ^E89G^) decreased DNA binding activity. Characterization of these mice revealed that, while each allele compromised the Notch pathway, they were insufficient to cause dominant phenotypes in an otherwise WT background. However, mice that were compound heterozygous for a *Notch1*-null allele and the *Rbpj^E89G^* allele had decreased viability and showed pronounced vascular and heart defects. In contrast, compound heterozygous mice with *Notch1-* and *Rbpj*-null alleles were born at normal Mendelian ratios and showed no gross morphological defects. These findings are consistent with AOS-associated *Rbpj* variants encoding dominant-negative proteins and not null alleles. Since an *Rbpj*-null allele is well tolerated in mice, we used conditional genetics to demonstrate that expressing the *Rbpj^E89G^* dominant-negative allele in endothelial cells is both necessary and sufficient to induce lethality due to vascular and heart-related defects. These studies provide mechanistic insights into how defective Notch signaling in the endothelium causes pathogenesis in mice and thereby serves as a useful model to study human AOS pathogenesis.

## Results

### AOS-associated RBPJ variants reduce DNA but not cofactor binding.

**Figure 1:**
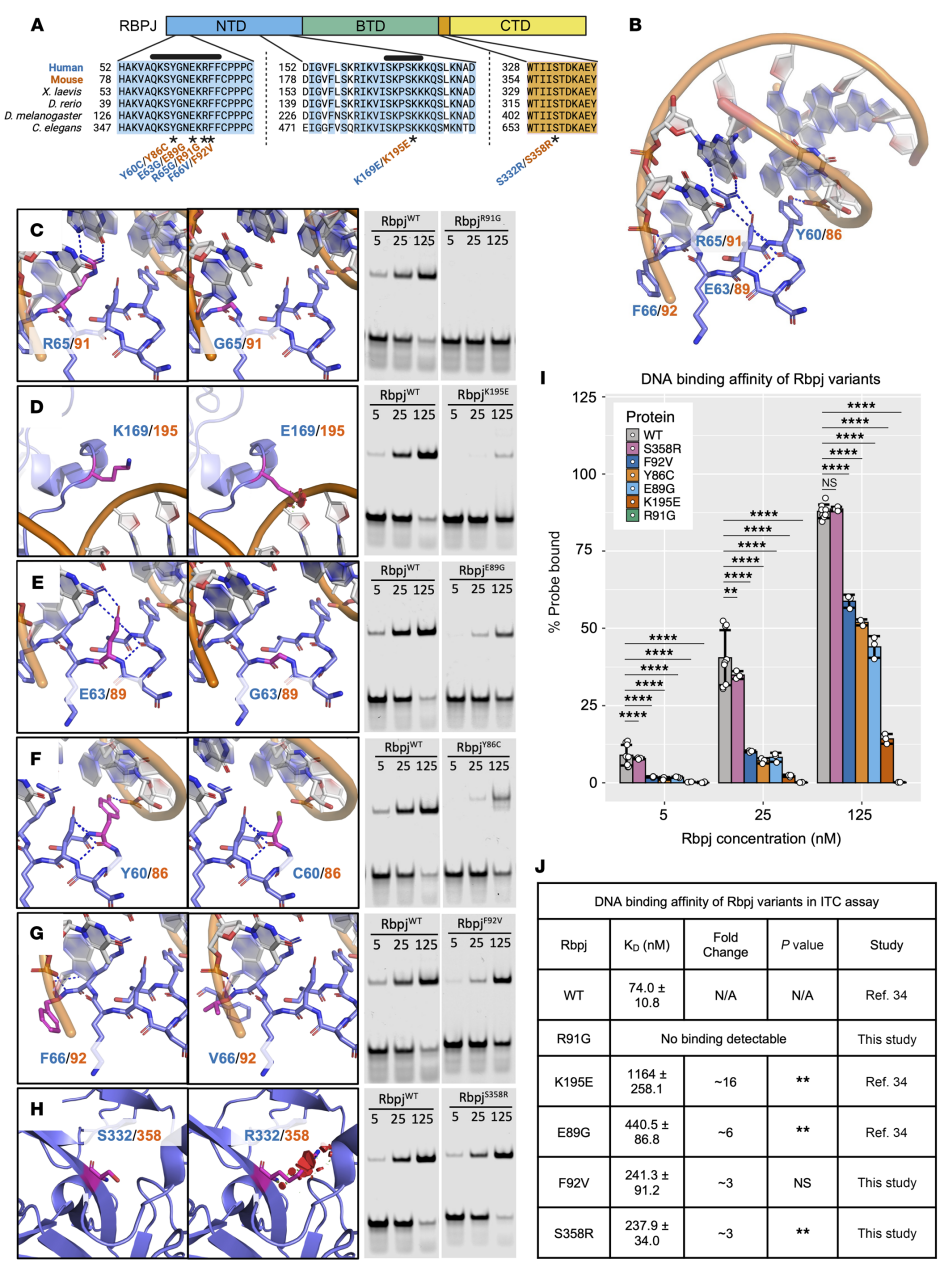
AOS-associated RBPJ variants impair DNA binding. (**A**) Domain map and sequence alignment of RBPJ orthologs. Conserved residues are highlighted, and AOS-associated variants (*) are denoted by human (blue) and mouse (orange) residue numbers. Black bars indicate DNA-binding regions. NTD = N-terminal domain. BTD = beta-trefoil domain. CTD = C-terminal domain. (Created in BioRender.) (**B**) Structure of RBPJ on DNA with AOS-associated residue changes denoted by human (blue) and mouse (orange) numbers. (**C**–**H**) PyMOL models of structural changes and representative comparative EMSAs of AOS-associated RBPJ variants. Dashed lines within each model denote DNA-residue or residue-residue polar interactions, and red discs indicate steric clash. EMSAs were performed using equimolar concentrations (5, 25, and 125 nM) of WT mouse RBPJ and the R91G (**C**), K195E (**D**), E89G (**E**), Y86C (**F**), F92V (**G**), and S358R (**H**) variants on a DNA probe encoding a high-affinity RBPJ binding site. (**I**) Graph quantifying the probe depletion for each variant across triplicate EMSAs (see Supplemental Figure 1). A 1-way ANOVA with Tukey’s post hoc correction was used to compare WT RBPJ with each variant. (**J**) Tabulated ITC data measuring DNA binding affinity of RBPJ variants. Fold-change calculated relative to WT RBPJ. A 2-tailed *t* test was used to compare *K*_D_ of WT RBPJ to each variant. *P < 0.05, **P < 0.01, ****P < 0.0001. NS, not significant. N/A, not applicable.

RBPJ has a conserved core consisting of an N-terminal domain (NTD), beta-trefoil domain (BTD), interdomain linker, and C-terminal domain (CTD) (Figure 1A). In the human ortholog (NM_005349.4), residues 57–67 and 165–170 in the NTD and 192–197 in the BTD directly interact with DNA (Figure 1, A and B) (36). To date, 6 likely deleterious RBPJ variants have been reported in AOS, all of which are missense substitutions that alter highly conserved residues (Y60C, E63G, R65G, F66V, K169E, and S332R; Figure 1, A and B) (2, 5). Five of these missense variants occur within the RBPJ DNA binding domain, whereas S332R occurs within the linker region (Figure 1, A and B). Consistent with the locations of these point mutations, prior studies characterized the DNA binding properties of two RBPJ disease variants (E63G and K169E) and found decreased DNA binding (5). These studies led to the prediction that AOS-associated RBPJ variants behave as loss-of-function alleles due to decreased DNA binding.

To determine whether all RBPJ AOS variants affect DNA binding and to directly compare the binding activity of each variant, we performed electrophoretic mobility shift assays (EMSAs) and isothermal titration calorimetry (ITC) assays using DNA probes encoding an RBPJ binding site and purified AOS-associated RBPJ variants within the context of the mouse protein (Figure 1 and Supplemental Figures 1 and 2; supplemental material available online with this article; https://doi.org/10.1172/JCI187532DS1). In addition, we modeled each variant in the context of the known RBPJ/DNA structure to better understand the molecular nature of each defect (Figure 1, C–H). Note, we previously reported ITC assays to assess the DNA binding affinity of WT RBPJ and the RBPJ^E89G^ and RBPJ^K195E^ AOS variants (34). We included that data here along with new EMSA data for comparative purposes, and we have cited the original source as appropriate. Collectively, these studies revealed 2 findings: first, all variants significantly decreased DNA binding compared with WT RBPJ; and second, the variants’ impact on DNA binding fell across a spectrum of severity (Figure 1, C–J, and Supplemental Figures 1 and 2). Below, we describe the impact of each variant.

The most severe variant was RBPJ^R91G^, which abolished DNA binding in EMSAs (Figure 1, C and I) and ITC assays (Figure 1J and Supplemental Figure 2A). This finding is congruent with the R91G change being predicted to abolish polar interactions with both DNA and the adjacent E89 residue (Figure 1C). Almost as severe was RBPJ^K195E^, which significantly compromised DNA binding in EMSAs (Figure 1, D and I) and decreased binding approximately 16-fold in ITC assays (Figure 1J and Supplemental Figure 2A). Consistent with this dramatic loss in DNA binding, the K195E change introduced electrostatic repulsion and steric clashing within a region involved in direct binding to the DNA backbone (Figure 1D).

The RBPJ^E89G^ and RBPJ^Y86C^ variants decreased DNA binding to a similar extent in EMSAs (Figure 1, E, F, and I). ITC assays further showed that RBPJ^E89G^ resulted in an approximately 6-fold loss in DNA binding relative to WT RBPJ (Figure 1J and Supplemental Figure 2A). Consistent with these findings, the E89G change is predicted to abolish polar interactions with Y86 and R91. Unfortunately, we were unable to purify sufficient RBPJ^Y86C^ to perform ITC assays. Moreover, the RBPJ^Y86C^/DNA complex migrated slower than WT RBPJ and all other tested variants in EMSAs, even though these proteins were similar in size in SDS gels (Supplemental Figure 1B). Since *Rbpj^Y86C^* introduces a Cys residue, we treated the protein with reducing agents and performed EMSAs but did not observe a change in this slower migration pattern (Supplemental Figure 1C). Although it is unclear why the Y86C substitution results in a slower migrating band, the similar loss of affinity observed by RBPJ^Y86C^ and RBPJ^E89G^ in EMSAs is consistent with structural analysis showing that Y86C is predicted to disrupt polar and nonpolar interactions with DNA (Figure 1F).

The last 2 variants, RBPJ^F92V^ and RBPJ^S358R^, resulted in weaker but still significant decreases in DNA binding in EMSAs compared with WT RBPJ (Figure 1, G and H). ITC assays confirmed an approximately 3-fold decrease in DNA binding affinity for each variant (Figure 1J and Supplemental Figure 2A). The modest impact on DNA binding is consistent with S358R residing in a region that does not directly contact DNA. However, this variant is predicted to induce steric clashing with surrounding residues (Figure 1H) and thereby could cause protein folding changes that result in decreased DNA binding. The F92V variant is not predicted to change polar interactions or introduce steric clashing. However, F92 appears to have substantial nonpolar interactions with the DNA backbone that the smaller V92 residue may not fully recapitulate (Figure 1G). Taken together, these DNA binding assays show that all RBPJ AOS variants negatively affect DNA binding but to varying degrees.

These DNA binding assays support the idea that *RBPJ* AOS alleles encode defective transcription factors that fail to properly bind DNA. In addition to binding DNA, RBPJ directly recruits NICD to activate transcription and corepressors to inhibit transcription. We previously showed that 2 AOS variants, RBPJ^E89G^ and RBPJ^K195E^, do not significantly alter their affinity for the NICD1 coactivator or the SHARP corepressor (34). Here, we found that RBPJ^F92V^ binds both NICD1 and SHARP with similar affinities as WT RBPJ and that RBPJ^R91G^ binds NICD1 with a similar affinity as WT RBPJ in ITC assays (Supplemental Figure 2, B and C, and Supplemental Table 1). Since Y86C is similarly found far from the NICD and SHARP interaction regions, this variant is also unlikely to alter cofactor binding. However, because S358R is located within a region not directly associated with DNA or cofactor binding, we tested RBPJ^S358R^ in ITC assays (Supplemental Figure 2, B and C) and found that it also binds NICD1 and SHARP with the same affinity as WT RBPJ (Supplemental Table 1). Thus, all RBPJ variants associated with AOS negatively affect DNA binding but not cofactor binding, consistent with the model that RBPJ AOS variants act as dominant-negative proteins that sequester cofactors away from WT RBPJ and off DNA.

### Rbpj^E89G^ and Rbpj^S358R^ mouse models reveal that phenotypic severity correlates with loss in DNA binding affinity.

**Figure 2:**
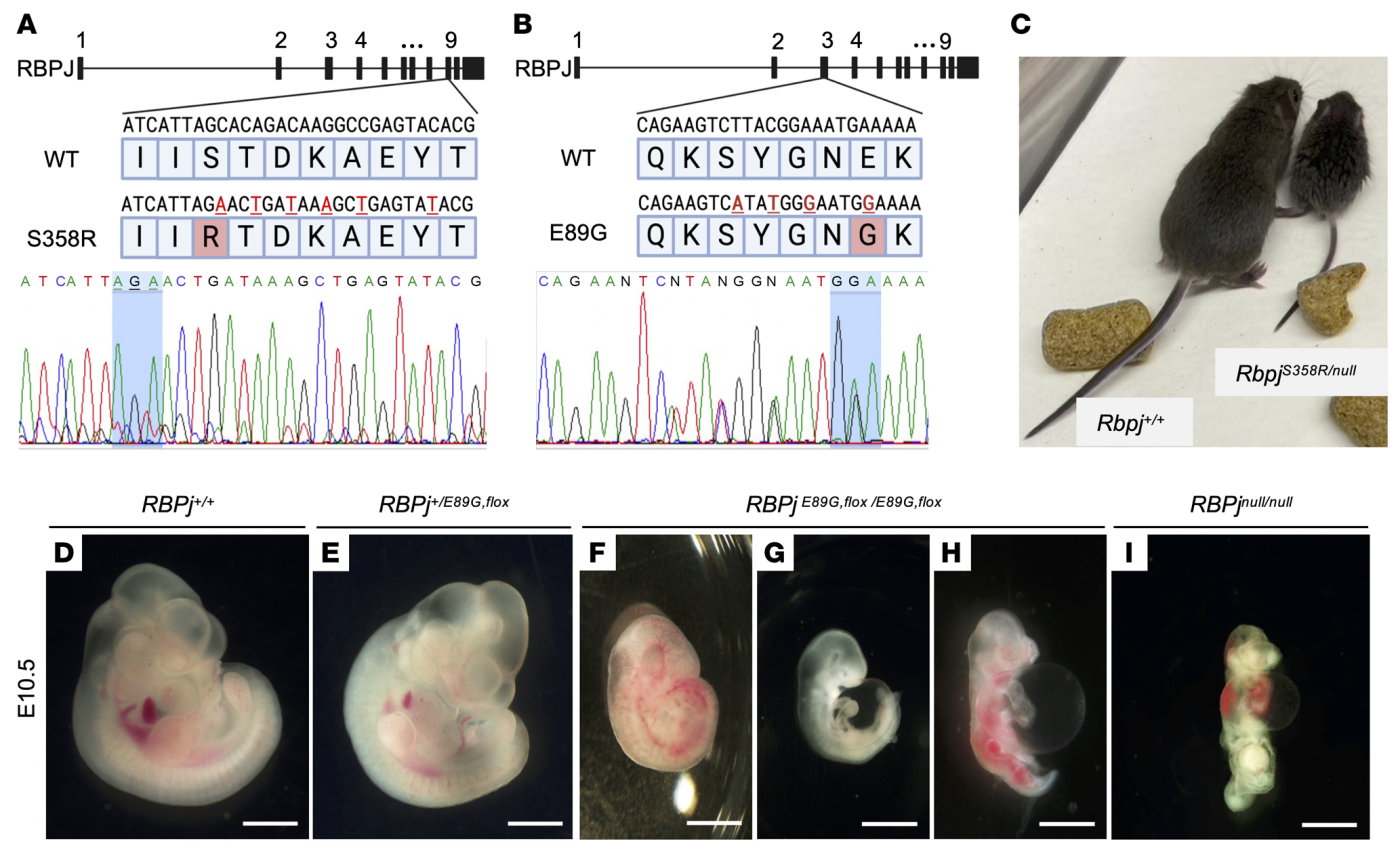
Generation of AOS-associated *Rbpj* variant mouse models reveals impaired animal growth and development. (**A**) (*top*) Schematic of mouse *Rbpj*, detailing the region of exon 9 encoding S358 and the donor sequence used to introduce the S358R substitution. (Created in BioRender.) (*bottom*) Confirmation of mouse genotype by Sanger sequencing with the codon for S358/R358 highlighted. (**B**) (*top*) Schematic of mouse *Rbpj*, detailing the region of exon 3 encoding E89 and the donor sequence used to introduce the E89G substitution. (Created in BioRender.) (*bottom*) Confirmation of mouse genotype by Sanger sequencing with the codon for E89/G89 highlighted. (**C**) Image showing that a typical P17 *Rbpj^S358R/null^* hemizygote (right) is much smaller than its *Rbpj^+/+^* littermate (left). (**D**–**I**) Stereoscope images of E10.5 embryos show that *Rbpj^E89G,fl/E89G,fl^* homozygotes (**F**–**H**) display growth retardation, hemorrhage, pallor, and/or pericardial edema of variable severity. *Rbpj^null/null^* homozygotes (**I**) show similar, albeit more severe, defects. Scale bar: 1 mm.

To make mouse models with AOS-associated *Rbpj* alleles, we used CRISPR/Cas9 gene editing to engineer 2 *Rbpj* mutations. We chose to model the *Rbpj^S358R^* and *Rbpj^E89G^* variants based on their mild (approximately 3-fold loss) and moderate (approximately 6-fold loss) impacts on DNA binding affinity, respectively, to avoid potential heterozygote lethality in a mouse carrying a severe variant. To introduce S358R (human S332R), we used a donor sequence to replace part of exon 9 of mouse *Rbpj* (Figure 2A). We similarly introduced E89G (human E63G) using a donor sequence to replace part of exon 3 (Figure 2B). In both cases, silent mutations were included to introduce restriction enzyme sites that facilitate genotyping, and each variant was confirmed by sequencing (Figure 2, A and B). Note, *Rbpj^S358R^* was generated on a WT *Rbpj* allele, and we created *Rbpj^E89G^* on the well-characterized *Rbpj^fl^* allele (37). Our rationale for making *Rbpj^E89G^* on the floxed allele is that Cre can be used to convert the dominant-negative *Rbpj^E89G,fl^* allele into an *Rbpj^null^* allele in select tissues of heterozygous mice that still have a non-floxed WT *Rbpj* allele (i.e., *Rbpj^+/E89G,fl^*).

To determine the impact of these *Rbpj* alleles on mouse viability, we assessed offspring for deviation from expected Mendelian ratios. These studies revealed that *Rbpj^+/S358R^* heterozygous and *Rbpj^S358R/S358R^* homozygous mice were viable and occurred at expected ratios (Table 1). Moreover, these mice did not show gross morphological defects, although *Rbpj^S358R/S358R^* mice were initially smaller than littermates but were of normal size by P5 (Supplemental Figure 3A). We subsequently crossed *Rbpj^S358R/S358R^* mice with mice carrying an *Rbpj*-null allele (*Rbpj^+/null^*) and found that *Rbpj^S358R/null^* hemizygotes had significantly reduced viability (Table 1), and surviving offspring were much smaller than littermates (Figure 2C and Supplemental Figure 3B). Thus, the *Rbpj^S358R^* allele behaves as a weak hypomorph in mice.

We similarly assessed the *Rbpj^E89G,fl^* allele and found that, although heterozygous mice (*Rbpj^+/E89G,fl^*) were viable and lacked gross morphological defects, no *Rbpj^E89G,fl/E89G,fl^* homozygotes were observed among live offspring (Table 1). To determine when *Rbpj^E89G,fl/E89G,fl^* homozygotes perish, we performed timed collections at E10.5. Although *Rbpj^+/E89G,fl^* embryos resembled WT littermates (Figure 2, D and E), we observed a lower-than-expected frequency of *Rbpj^E89G,fl/E89G,fl^* embryos (Table 1), and all homozygous embryos were much smaller than their littermates (Figure 2, F–H). Western blot analysis of protein isolated from E10.5 *Rbpj^E89G,fl/E89G,fl^* and WT embryos revealed that RBPJ^E89G^ was expressed at normal levels relative to β-actin (Supplemental Figure 4), consistent with prior studies showing that RBPJ^E89G^ had similar stability as WT RBPJ in cell culture (34). Visual analysis of these embryos revealed a range of morphological defects that included hemorrhages (Figure 2F, *n* = 4/8), pericardial edema (Figure 2, G and H, *n* = 6/8), pallor (Figure 2G, *n* = 3/8), and incomplete axial rotation (Figure 2H, *n* = 3/8). The pericardial edema and incomplete axial rotation are reminiscent of *Rbpj^null/null^* embryos (Figure 2I), although *Rbpj^E89G,fl/E89G,fl^* embryos fare slightly better than *Rbpj^null/null^* embryos. Lastly, we crossed *Rbpj^+/E89G,fl^* mice with *Rbpj^+/S358R^* mice and observed a dramatic loss of viability in offspring with both the *Rbpj^S358R^* and *Rbpj^E89G,fl^* alleles (*Rbpj^S358R/E89G,fl^*, Table 1). Altogether, these data show that the RBPJ^E89G^ variant, which has an approximately 6-fold decrease in DNA binding activity, causes more severe phenotypes in mice than the RBPJ^S358R^ variant with an approximately 3-fold loss in DNA binding.

### A compound heterozygous mouse model carrying Rbpj^E89G^ and N1^null^ AOS alleles has vascular and heart phenotypes.

**Figure 3:**
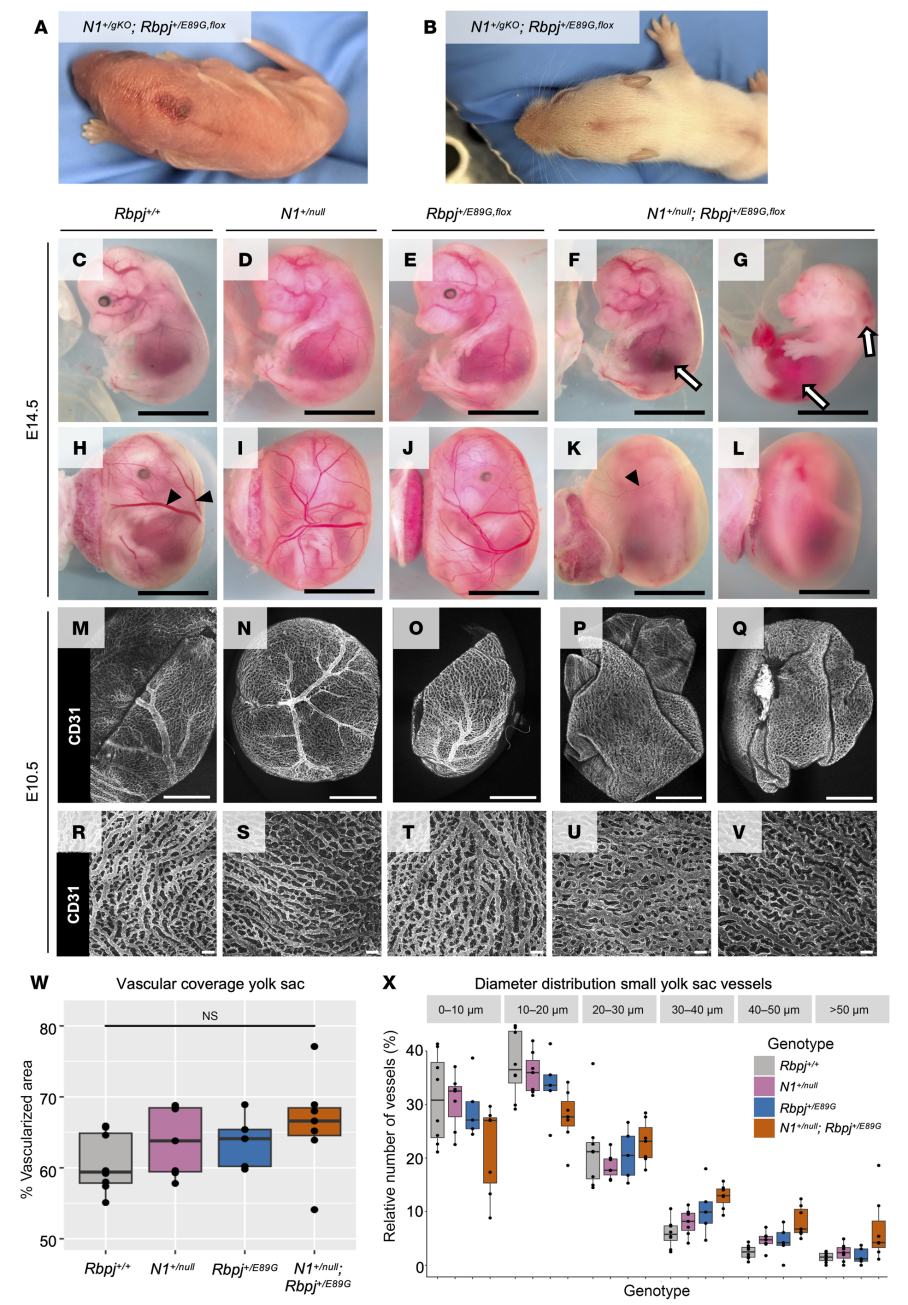
*N1^+/null^ Rbpj^+/E89G,fl^* embryos display vascular phenotypes. (**A** and **B**) Representative images of dorsal midline skin lesions in P0 (**A**) and P11 (**B**) *N1^+/gKO^ Rbpj^+/E89G,fl^* mice. (**C**–**G**) Representative images of E14.5 embryos for WT (*Rbpj^+/+^*), *N1^+/null^*, and *Rbpj^+/E89G,fl^* single heterozygotes and *N1^+/null^ Rbpj^+/E89G,fl^* compound heterozygotes. Note, areas of hemorrhage (arrows) are observed in E14.5 *N1^+/null^ Rbpj^+/E89G,fl^* embryos but not in control embryos. (**H**–**L**) Representative images of E14.5 embryos within their yolk sac for the indicated genotypes. Note, the compound heterozygous embryos have reduced or absent yolk sac vasculature (filled arrowheads). (**M**–**Q**) Representative 4× original magnification images of CD31-stained yolk sacs from E10.5 embryos for the indicated genotypes. (**R**–**V**) Representative 10× original magnification images of CD31-stained yolk sac microvasculature from E10.5 embryos for indicated genotypes. Scale bars: 0.5 cm (**C**–**L**), 1 mm (**M**–**Q**), and 100 μm (**R**–**V**). (**W**) Percentage of vascular coverage of yolk sacs measured in representative areas for 5–7 embryos per each indicated genotype. Each dot represents the yolk sac from an individual embryo, and the box plot shows the median with the 25th and 75th quartile highlighted. (**X**) Distribution of vessels by diameter using representative 400 μm × 400 μm areas of the yolk sac capillary networks stained for CD31. Vessel diameters were assessed between all branch points and measured using the NIS-Elements measurements tool. Each dot represents the yolk sac from an individual embryo, and the box plot shows the median with the 25th and 75th quartile highlighted.

Our data with the *Rbpj^S358R^* and *Rbpj^E89G,fl^* alleles revealed that neither was sufficient to cause dominant AOS-like phenotypes. In contrast, patients heterozygous for analogous *RBPJ* variants have dominant AOS phenotypes, although the *RBPJ^S332R^* allele shows incomplete penetrance with only a single symptomatic patient and nonsymptomatic parent (2). These findings are consistent with prior studies showing differences in sensitivity to Notch pathway alleles between mice and humans. For example, *NOTCH1* haploinsufficiency can cause human disease such as AOS and aortic valve disease (25), whereas a *Notch1-*null (*N1*-null) allele is well tolerated in heterozygous mice (33, 38). Interestingly, a family with AOS was found to have compound heterozygous mutations in both *RBPJ* and *NOTCH1* alleles (2). Hence, we crossed *Rbpj^+/E89G,fl^* mice with mice heterozygous for either an *N1*-null allele that deletes amino acids 1056–2049, thereby removing several EGF repeats, the transmembrane domain, and Ankyrin repeats (*N1^tm1Con^*; ref. 38; referred to here as *N1^null^*), or an *N1*-null allele that deletes the promoter and exon 1 (*N1^tm2Agt^*; ref. 39; referred to here as *N1^gKO^*). Importantly, we observed a dramatic loss of viability in both *N1^+/null^ Rbpj^+/E89G,fl^* and *N1^+/gKO^ Rbpj^+/E89G,fl^* compound heterozygous mice (Table 2), and the surviving mice generally failed to thrive. Intriguingly, a subset of the *N1^+/gKO^ Rbpj^+/E89G,fl^* mice, which had considerable C57/BL6 in their background, had obvious morphological skin/scalp defects (Figure 3, A and B). These findings raise the possibility of genetic background contributing to the skin/scalp defect. Hence, in this study, we focused on identifying the mechanisms of embryonic lethality, which was observed with both *N1* alleles in outbred backgrounds.

We next assessed the specificity of the genetic interactions between *N1* and *Rbpj^E89G^* by performing 2 additional tests. First, we crossed each *N1*-null allele with mice carrying an *Rbpj*-null allele and found that neither *N1^+/null^ Rbpj^+/null^* nor *N1^+/gKO^ Rbpj^+/null^* were significantly underrepresented (Table 2). Moreover, unlike the *N1* and *Rbpj^+/E89G,fl^* compound heterozygotes that showed morphological defects and failed to thrive, the *N1^+/null^ Rbpj^+/null^* and *N1^+/gKO^ Rbpj^+/null^* compound heterozygous mice were indistinguishable from littermate controls. Thus, the decreased viability observed in the *N1* and *Rbpj^+/E89G,fl^* compound heterozygotes was due to the presence of the *Rbpj^E89G,fl^* allele and not simply due to loss of a WT *Rbpj* allele. Second, we crossed the *Rbpj^+/E89G,fl^* allele into a *Notch2*-sensitized *(N2*-sensitized) background and observed expected numbers of *N2^+/lacZ^ Rbpj^+/E89G,fl^* compound heterozygotes that showed no gross morphological defects (Table 2). Thus, the *Rbpj^E89G^* allele genetically interacts with *N1*-null alleles to cause decreased viability but not with an *N2*-null allele. These data are consistent with clinical findings showing that RBPJ variants cause a NOTCH1-like syndrome (AOS) but not a NOTCH2-like syndrome (Alagille syndrome) (25, 40).

The decreased viability and failure of *N1^+/null^ Rbpj^+/E89G,fl^* mice to thrive made it difficult to obtain sufficient mice to perform quantitative analyses of postnatal tissues. To define the cause of lethality in *N1^+/null^ Rbpj^+/E89G,fl^* compound heterozygotes, we first genotyped embryos from timed harvests at E10.5, E14.5, and E16.5 to assess the time of embryonic demise. These experiments revealed a gradual decrease in *N1^+/null^ Rbpj^+/E89G,fl^* compound heterozygous embryos that became significant by E16.5 (Table 2). Moreover, gross morphological analysis of these embryos revealed vascular phenotypes that included hemorrhages (Figure 3, C–G) and a dramatic reduction in large vessels within the yolk sac vasculature (Figure 3, H–L). Since loss of large vessels could be caused by a lack of vascular remodeling, we stained yolk sacs from E10.5 embryos for the endothelial marker CD31 (Figure 3, M–V). Low magnification images confirmed an overall decrease in large vessels within the yolk sacs of *N1^+/null^ Rbpj^+/E89G,fl^* embryos (Figure 3, P and Q) compared with single heterozygous and WT littermates (Figure 3, M–O). However, higher magnification images revealed a robust network of yolk sac capillary vessels in all embryos including *N1^+/null^ Rbpj^+/E89G,fl^* compound heterozygotes (Figure 3, R–V). This capillary bed initially forms via vasculogenesis prior to E8.5 and then undergoes N1-dependent remodeling between E8.5 and E10.5 to form a branched hierarchical network of large and small vessels (41). Comparative analysis of the capillary bed revealed that, while the WT and single heterozygous yolk sac vessels had successfully undergone remodeling to form a network of different sized vessels (Figure 3, R–T), the *N1^+/null^ Rbpj^+/E89G,fl^* compound heterozygotes showed a range of phenotypes consistent with a lack of or partial failure to undergo hierarchical vascular patterning (Figure 3, U and V, respectively). We next quantified the percentage of vascularized area and the diameter distribution of capillary vessels in the yolk sacs from at least 5 embryos per genotype. Although this analysis revealed that, as a group, the *N1^+/null^ Rbpj^+/E89G,fl^* yolk sac capillary bed vasculature was not significantly different from littermate controls (Figure 3, W and X), the *N1^+/null^ Rbpj^+/E89G,fl^* embryos showed greater phenotype variability than control embryos. These data are consistent with *N1^+/null^ Rbpj^+/E89G,fl^* compound heterozygotes having a partially penetrant disruption or delay in remodeling of the early vascular plexus.

**Figure 4:**
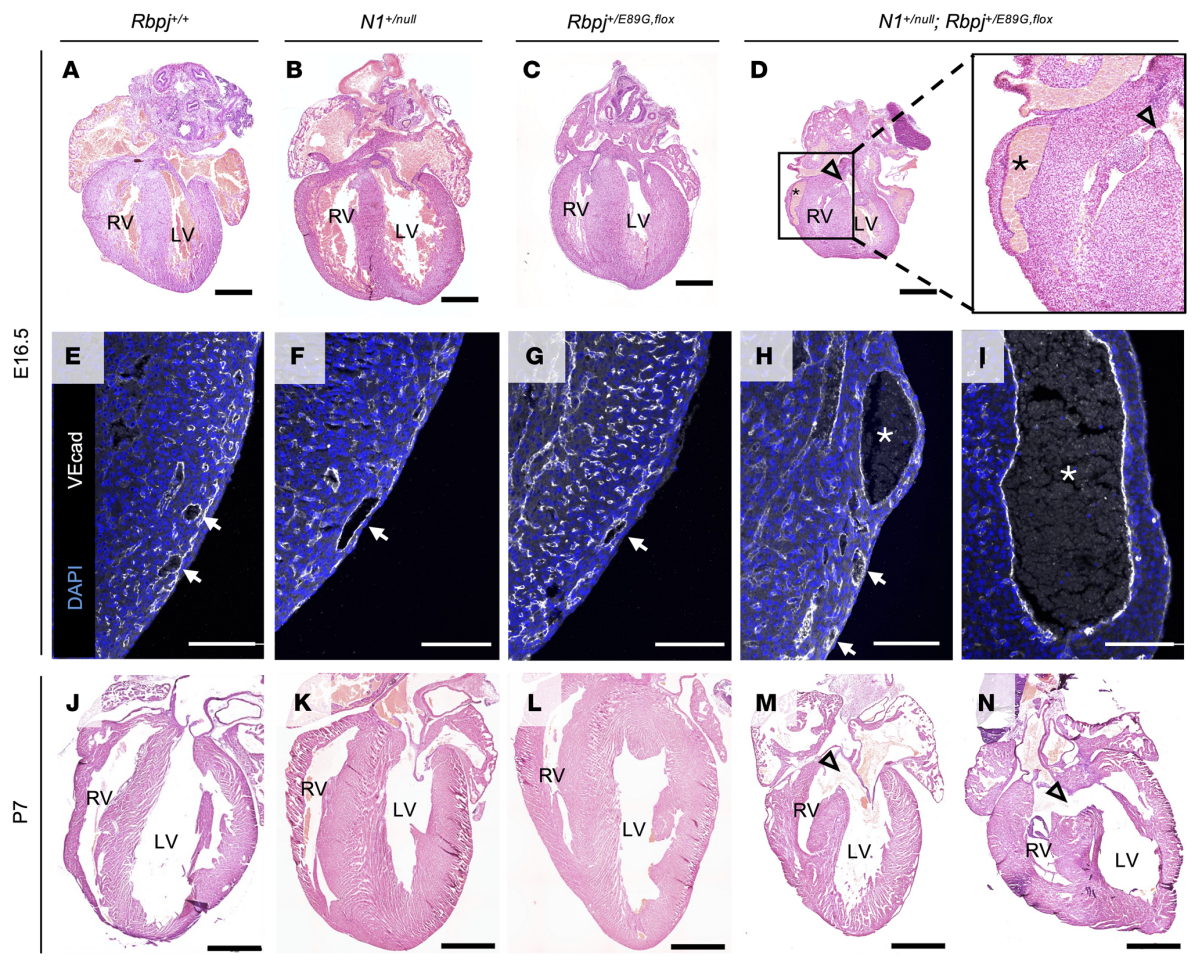
*N1^+/null^ Rbpj^+/E89G,fl^* embryos display cardiac phenotypes. (**A**–**D**) Representative images of E16.5 H&E-stained heart sections from WT (*Rbpj^+/+^*), *N1^+/null^*, *Rbpj^+/E89G,fl^*, and *N1^+/null^ Rbpj^+/E89G,fl^* genotypes. The left ventricle (LV) and right ventricle (RV) are labeled, and arrowheads highlight ventricular septal defects in the *N1^+/null^ Rbpj^+/E89G,fl^* heart, whereas asterisks highlight dilated coronary vessels. The box in **D** outlines the region shown at higher magnification at left. (**E**–**I**) Representative images of E16.5 heart sections that were stained with VE-cadherin (endothelium, white) and DAPI (nuclei, blue). Arrows indicate coronary vessels, with the lumens of dilated vessels indicated with asterisks. (**J**–**N**) Representative images of P7 H&E-stained heart sections from WT (*Rbpj^+/+^*), *N1^+/null^*, *Rbpj^+/E89G,fl^*, and *N1^+/null^ Rbpj^+/E89G,fl^* genotypes. The left ventricle (LV) and right ventricle (RV) are labeled, and arrowheads highlight ventricular septal defects in *N1^+/null^ Rbpj^+/E89G,fl^* hearts. Scale bars: 0.5 mm (**A**–**D**), 100 μm (**E**–**I**), and 1 mm (**J**–**N**).

Since heart defects are common in both humans and mice with Notch pathway mutations, we analyzed E16.5 hearts and observed malformations that included ventricular septal defects (VSDs) and dilated coronary vessels in *N1^+/null^ Rbpj^+/E89G,fl^* embryos (Figure 4, A–D; we quantify these defects below). We confirmed that the dilated structures in *N1^+/null^ Rbpj^+/E89G,fl^* hearts were blood vessels using the endothelial marker VE-cadherin (Fih–I). Consistent with these data, analysis of the hearts from the relatively few P7 *N1^+/null^ Rbpj^+/E89G,fl^* mice revealed that one-third also had VSDs (2 of 6, Figure 4, J–N). Although *NOTCH1* variants in humans have been associated with bicuspid valve disease, we did not observe obvious valve abnormalities in the hearts of either E16.5 or P7 *N1^+/null^ Rbpj^+/E89G,fl^* animals. Altogether, these data demonstrated that *N1^+/null^ Rbpj^+/E89G,fl^* mice show increased embryonic lethality that is potentially caused by hemorrhages, diminished yolk sac vascular remodeling, and/or cardiovascular defects.

### Conditional removal of the Rbpj^E89G,fl^ allele from only endothelial cells rescues cardiovascular phenotypes.

**Figure 5:**
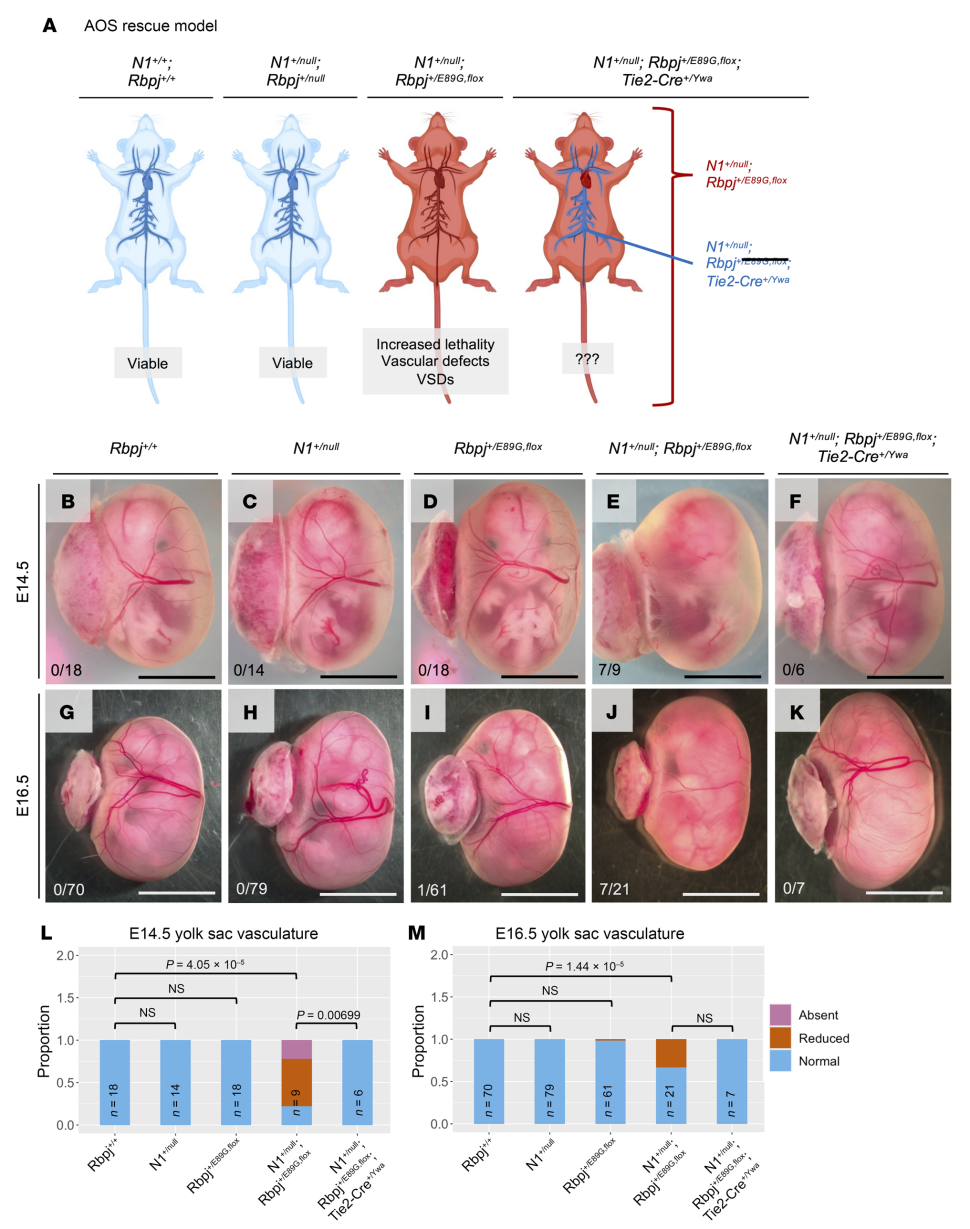
Conditional removal of *Rbpj^E89G^* from the endothelium rescues vascular phenotypes. (**A**) Schematic of AOS rescue model. Both WT (*N1^+/+^ Rbpj^+/+^*) and *N1^+/null^ Rbpj^+/null^* mice are viable and without overt defects. Mice with the *N1^+/null^ Rbpj^+/E89G,fl^* genotype have reduced viability, vascular defects, and heart defects (see Table 3 and Figures 3 and 4). A mouse that recombines *N1^+/null^ Rbpj^+/E89G,fl^* to *N1^+/null^ Rbpj^+/null^* in the endothelium using *Tie2-Cre^Ywa^* tests the necessity of the variant in the vascular endothelium for the development of AOS-like phenotypes. (Created in BioRender.). (**B**–**K**) Representative images of E14.5 embryos (**B**–**F**) and E16.5 embryos (**G**–**K**) within their yolk sac for the indicated genotypes. Note, only the *N1^+/null^ Rbpj^+/E89G,fl^* embryos have reduced or absent yolk sac vasculature. The ratio of affected to total individuals is listed in the lower left corner of each panel. (**L** and **M**) Visualization of the proportion of embryos with yolk sac vasculature defects at each stage. *P* values calculated with Fisher’s exact test are noted; NS, not significant.

Two pieces of evidence have led to the hypothesis that AOS is largely a vascular disease. First, patients with AOS with *NOTCH1*, *DLL4*, and *RBPJ* variants frequently have cardiovascular defects (2). Second, mouse and zebrafish studies have shown that N1 and DLL4 signaling are critical regulators of vascular development (25, 26). To test this hypothesis, we developed a conditional AOS “rescue” model that uses *Tie2-Cre^Ywa^* to specifically recombine floxed alleles in the developing endothelium (42), which includes the vascular endothelial cells that form the inner lining of blood vessels and the endocardial cells that line the heart. *Tie2* is not active in lymphatic endothelial cells, but it is active in hematopoietic stem cells (43). By crossing *N1^+/null^ Tie2-Cre^+/Ywa^* mice with *Rbpj^+/E89G,fl^* mice, Cre recombination converts the floxed *Rbpj^E89G,fl^* allele into an *Rbpj^null^* allele in heterozygous endothelial cells and hematopoietic stem cells that still encode a WT *Rbpj^+^* allele (see schematic in Figure 5A). Since *N1^+/null^ Rbpj^+/null^* mice occur in expected numbers (Table 2) and do not show overt phenotypes, this mouse model explicitly tests whether expressing the *Rbpj^+/E89G,fl^* allele within endothelial cells and hematopoietic stem cells is required (i.e., necessary) to induce morbidity in an *N1^+/null^* background (Figure 5A). Consistent with this idea, *N1^+/null^ Rbpj^+/E89G,fl^* *Tie2-Cre^+/Ywa^* mice had significantly enhanced viability compared with *N1^+/null^ Rbpj^+/E89G,fl^* littermates that lack *Tie2-Cre* (Table 3). Moreover, the *N1^+/null^ Rbpj^+/E89G,fl^* *Tie2-Cre^+/Ywa^* mice were indistinguishable from control littermates (Supplemental Figure 5), whereas *N1^+/null^ Rbpj^+/E89G,fl^* mice without *Tie2-Cre* generally failed to thrive (Table 3). Thus, *Tie2-Cre* can significantly rescue the lethality seen in *N1^+/null^ Rbpj^+/E89G,fl^* mice by converting the *Rbpj^+/E89G,fl^* AOS allele into an *Rbpj^+/null^* allele within the endothelium.

Because few *N1^+/null^ Rbpj^+/E89G,fl^* mice without *Tie2-Cre* survive postnatally, we quantified the impact of converting the *Rbpj^+/E89G,fl^* allele into an *Rbpj^+/null^* allele using timed embryo collections at E14.5 and E16.5. Consistent with our postnatal analysis, *Tie2-Cre* was sufficient to rescue lethality of *N1^+/null^ Rbpj^+/E89G,fl^* embryos at E16.5, whereas *N1^+/null^ Rbpj^+/E89G,fl^* littermates without *Tie2-Cre* were significantly underrepresented (Table 3). Moreover, analysis of the yolk sac at both E14.5 and E16.5 revealed that *Tie2-Cre* significantly rescued the vascular defects of *N1^+/null^ Rbpj^+/E89G,fl^* embryos (Figure 5, B–K). For example, although 7 of 9 E14.5 *N1^+/null^ Rbpj^+/E89G,fl^* embryos had reduced or absent yolk sac vasculature, 0 of 6 E14.5 *N1^+/null^ Rbpj^+/E89G,fl^ Tie2-Cre^+/Ywa^* embryos and none of the control littermates showed diminished yolk sac vasculature (Figure 5, B–F, and L). A similar rescue in yolk sac vasculature was observed in *Tie2-Cre* positive *N1^+/null^ Rbpj^+/E89G,fl^* embryos at E16.5 (Figure 5, G–K, and M). Thus, conditionally converting *Rbpj^+/E89G,fl^* into an *Rbpj^+/null^* allele with *Tie2-Cre* was sufficient to rescue both viability and yolk sac vasculature defects in *N1^+/null^* heterozygous embryos. Intriguingly, comparative analysis between embryonic time points revealed that the penetrance of yolk sac vasculature defects in the absence of *Tie2-Cre* was significantly decreased at E16.5 (approximately 33%) compared with E14.5 (approximately 78%) in *N1^+/null^ Rbpj^+/E89G,fl^* embryos (*P* = 0.046). This decreased penetrance in older embryos correlates well with the viability data showing a decrease in the proportion of *N1^+/null^ Rbpj^+/E89G,fl^* embryos from E14.5 to E16.5 (Table 2). Hence, these data suggest that those E14.5 embryos with severe yolk sac phenotypes are likely to perish prior to E16.5 and that conditionally deleting the *Rbpj^+/E89G,fl^* allele using *Tie2-Cre* can rescue this phenotype and lethality.

To further assess for possible vascular defects, we immunostained the skin vasculature from the forelimb and scalp regions of E14.5 embryos using a CD31 antibody to label endothelial cells. Analysis of the forelimb tissues for both percentage of vascularized area and branch point density did not reveal significant differences across genotypes (Supplemental Figure 6, A–D). In addition, we analyzed tip cell numbers within the scalp vasculature at E14.5, a time point at which sprouting angiogenesis is actively occurring at the top of the skull, and did not observe any obvious changes in tip cell numbers across genotypes (Supplemental Figure 6, E–I). Thus, although significant defects in the yolk sac vasculature were observed in *N1^+/null^ Rbpj^+/E89G,fl^* embryos, we did not observe obvious widespread vascular defects within the embryonic skin.

**Figure 6:**
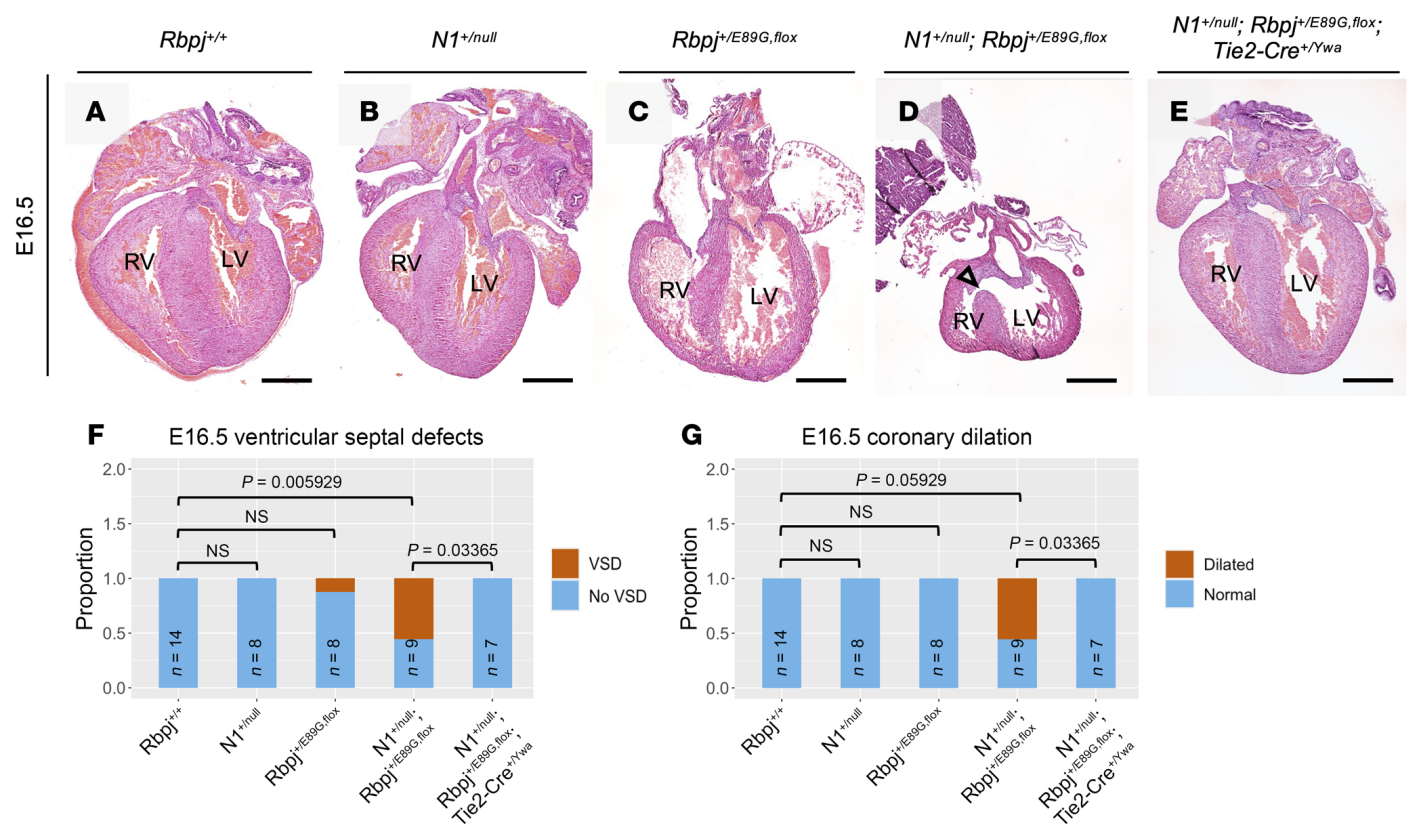
Conditional removal of *Rbpj^E89G^* from the vascular endothelium rescues heart phenotypes. (**A**–**E**) Representative images of E16.5 H&E-stained heart sections. The left ventricle (LV) and right ventricle (RV) are labeled, and an arrowhead highlights a ventricular septal defect in the *N1^+/null^ Rbpj^+/E89G,fl^* heart. (**F**–**G**) Visualization of the proportion of E16.5 embryos with (**F**) ventricular septal defects and (**G**) dilated coronary vessels. *P* values calculated with Fisher’s exact test are noted. ns = not significant.

Next, we assessed whether *Tie2-Cre* could rescue the heart defects seen in *N1^+/null^ Rbpj^+/E89G,fl^* embryos (see Figure 4). Unlike WT embryos (Figure 6A), *N1^+/null^* single heterozygotes (Figure 6B), and *Rbpj^+/E89G,fl^* single heterozygotes (Figure 6C), *N1^+/null^ Rbpj^+/E89G,fl^* compound heterozygotes showed heart defects at E16.5 that included VSDs (5 of 9, Figure 6, D and F) and coronary vessel dilation (5 of 9, Figure 6G). In contrast, we did not observe these phenotypes in *N1^+/null^ Rbpj^+/E89G,fl^* *Tie2-Cre^+/Ywa^* embryos (Figure 6, E–G), suggesting that the heart and vessel dilation defects in *N1^+/null^ Rbpj^+/E89G,fl^* embryos are due to compromised N1 signaling in the developing endothelial and endocardial cells. Together, these results show that expressing the AOS-associated dominant-negative RBPJ protein in the vascular endothelium is necessary to cause cardiovascular phenotypes.

### Selective induction of N1^+/cKO^ Rbpj^+/E89G^ compound heterozygosity in the vascular endothelium is sufficient to cause lethality and cardiovascular phenotypes.

**Figure 7:**
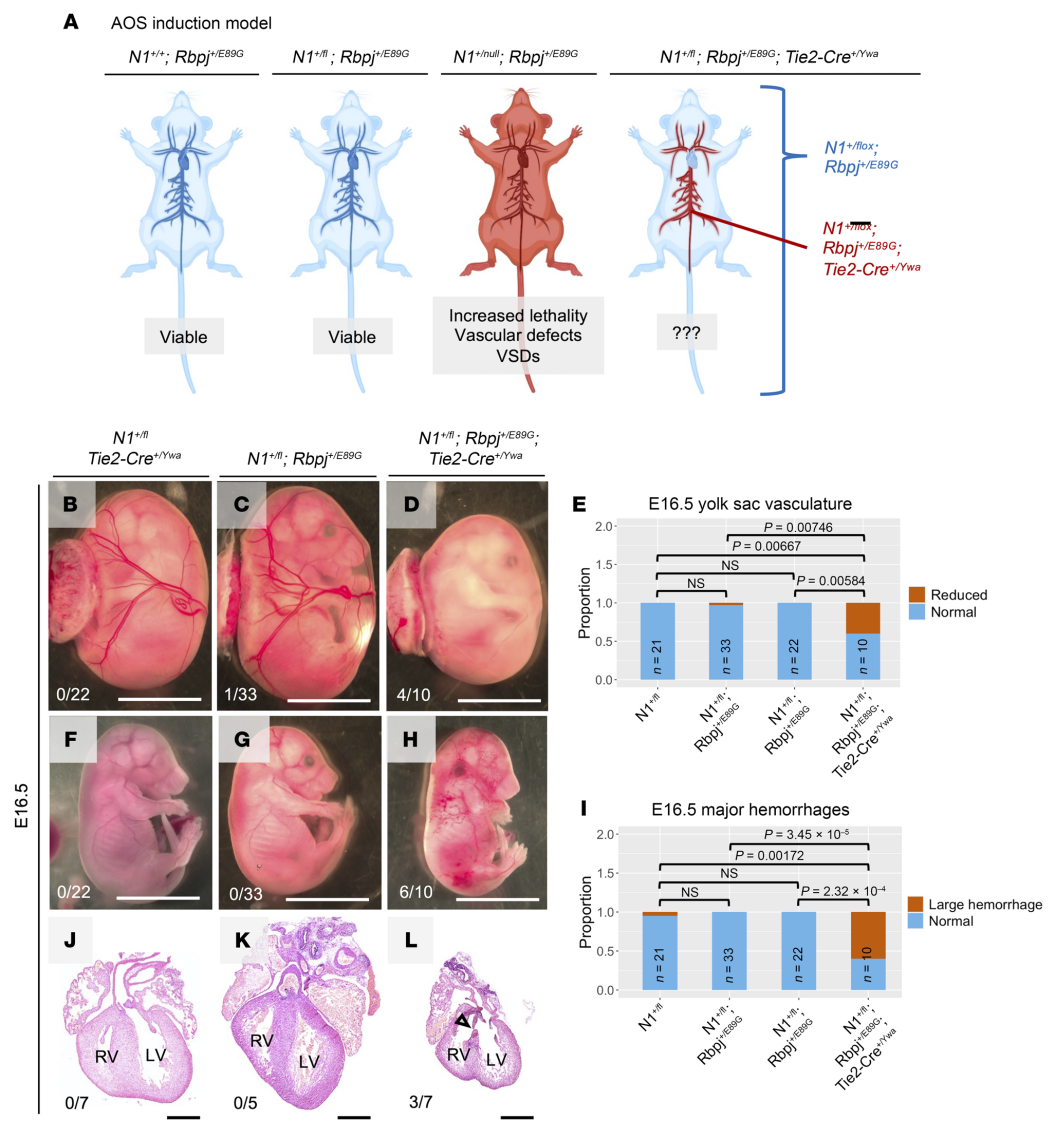
Conditional removal of one copy of *Notch1* from the vascular endothelium of *Rbpj^+/E89G^* mice induces vascular and heart phenotypes. (**A**) Schematics of AOS induction model. Both WT (*N1^+/+^ Rbpj^+/+^*) and *N1^+/fl^ Rbpj^+/E89G^* mice are viable and without overt defects (see Table 4). A mouse that recombines *N1^+/fl^ Rbpj^+/E89G^* to *N1^+/cKO^ Rbpj^+/E89G^* in the endothelium using *Tie2-Cre^Ywa^* tests the sufficiency of the variant’s presence in the vascular endothelium for the development of AOS-like phenotypes. (Created in BioRender.) (**B**–**E**) E16.5 *N1^+/fl^ Rbpj^+/E89G^ Tie2-Cre^+/Ywa^* embryos have reduced yolk sac vasculature, increased frequency of hemorrhage (**F**–**I**), and ventricular septal defects (**J**–**L**). The left ventricle (LV) and right ventricle (RV) are labeled, and an arrowhead highlights a ventricular septal defect in the *N1^+/fl^ Rbpj^+/E89G^ Tie2-Cre^+/Ywa^* heart. The ratio of affected individuals to total individuals is listed in the lower left corner of each panel. Scale bars: 0.5 cm (**B**–**D** and **F**–**H**) and 0.5 mm (**J**–**L**). *P* values calculated with Fisher’s exact test are noted; NS, not significant.

The AOS rescue model reveals that expressing *Rbpj^E89G^* in the endothelium is necessary to induce morbidity in *N1^+/null^* mice. To test whether expressing these alleles within only the endothelium and hematopoietic stem cells is sufficient to induce morbidity, we modified our conditional approach to create an AOS induction model (Figure 7A). First, we used genome editing to remake the *Rbpj^E89G^* variant on a non-floxed *Rbpj* allele. *Rbpj^+/E89G^ Tie2-Cre^+/Ywa^* mice were then crossed with *N1^fl/fl^* mice (44) to generate *N1^+/fl^Rbpj^+/E89G^* offspring with and without *Tie2-Cre*. In this model, *Tie2-Cre* selectively recombines the *N1^fl/fl^* allele into a null allele (*N1^cKO^*) to induce *N1^+/cKO^ Rbpj^+/E89G^* compound heterozygosity within endothelial cells and hematopoietic stem cells of mice that otherwise have 2 copies of *N1* (i.e., *N1^+/fl^ Rbpj^+/E89G^*) (Figure 7A). Consistent with our hypothesis, *N1^+/fl^ Rbpj^+/E89G^ Tie2-Cre^+/Ywa^* mice occurred significantly less often than their littermates, suggesting prenatal demise (Table 4). Moreover, E16.5 *N1^+/fl^ Rbpj^+/E89G^ Tie2-Cre^+/Ywa^* embryos had both significantly reduced yolk sac vasculature (Figure 7, B–E) and increased incidences of hemorrhage (Figure 7, F–I) compared with littermates. Additionally, VSDs were observed in *N1^+/fl^ Rbpj^+/E89G^ Tie2-Cre^+/Ywa^* hearts but not in control littermates (3 of 7, Figure 7, J–L). Thus, *N1^+/null^ Rbpj^+/E89G^* compound heterozygosity in the vascular endothelium is sufficient to cause lethality and cardiovascular defects.

## Discussion

In this study, we investigated mechanisms underlying how AOS-associated RBPJ variants cause pathogenesis. At the molecular level, we used DNA and protein-protein interaction assays to show that all known AOS-associated RBPJ variants reduce binding to DNA but not to the NICD1 coactivator nor the SHARP corepressor. These in vitro findings are supported by previous co-IP assays showing that full-length NICD1, MAML, and SHARP proteins interact similarly with WT RBPJ and 2 AOS variants (RBPJ^E89G^ and RBPJ^K195E^) and that RBPJ^E89G^ and RBPJ^K195E^ were both properly localized to the nucleus and had similar turnover rates as WT RBPJ (34). At the transcription level, however, titration of a DNA binding–deficient RBPJ variant into cells expressing WT RBPJ lowered Notch-mediated activation, whereas titrating in an RBPJ variant that could neither bind DNA nor NICD1 did not affect transcriptional activation (34). Moreover, a genomic and single-molecule study found that the RBPJ^K195E^ AOS variant bound significantly fewer genomic sites and had significantly shorter residency time on DNA than WT RBPJ in HeLa cells (45). Altogether, these biochemical and cellular data support a model whereby AOS-associated RBPJ variants dysregulate Notch signaling by competing for cofactors with WT RBPJ and sequestering them off DNA.

The idea that AOS RBPJ variants act as dominant-negative alleles is further supported by genetic studies. In *Drosophila*, we previously found that an analogous AOS mutation in the fly RBPJ homologue *Su(H)* causes dominant Notch phenotypes not observed in flies heterozygous for a *Su(H)*-null allele (34). Here, we similarly found that mice heterozygous for the *Rbpj^E89G^* AOS allele suffer lethality and cardiovascular defects in a sensitized *N1* background, whereas compound heterozygotes for *N1* and an *Rbpj*-null allele occur in normal ratios and suffer no obvious defects. Lastly, studies of patients with AOS identified 6 missense variants with decreased DNA binding, whereas no mutations have been identified that would render *RBPJ* into a null allele (2, 5). Moreover, a seventh AOS variant that affects R65 (R65T) was recently reported on ClinVar (VCV001803755.1; https://www.ncbi.nlm.nih.gov/clinvar/), and this variant is likely to negatively affect DNA binding in a manner similar to R65G. Interestingly, however, even though *RBPJ*-null alleles have not been implicated in AOS, they are underrepresented in the Genome Aggregation Database (pLI = 1; gnomAD v4.1.0) (46). This finding suggests *RBPJ* haploinsufficiency is likely deleterious in humans, and future studies are needed to determine the impact *RBPJ* haploinsufficiency has on human development.

Our comparative studies revealed that, while all 6 RBPJ variants compromise DNA binding, they do so to different degrees. These findings predict that RBPJ variants that more strongly decrease DNA binding will result in greater Notch dysregulation and worse outcomes. Consistent with this idea, mice with the RBPJ^E89G^ variant that decreases DNA binding 6-fold resulted in more severe phenotypes than mice with the RBPJ^S358R^ variant that decreases DNA binding 3-fold. Similarly, the *Drosophila* *Su(H)^T4^* allele that compromises DNA binding approximately 5-fold resulted in more severe Notch pathway dysregulation compared with the *Su(H)^O5^* allele encoding a protein with approximately 3.5-fold decreased DNA binding (34). Although the rarity of human AOS makes it difficult to perform a comprehensive comparison between variant DNA binding and clinical severity, it is interesting to note that the 2 variants with the weakest impact on DNA binding were found to either have incomplete penetrance (*RBPJ^S332R^*) or were only found in patients who carried both an *RBPJ^F66V^* allele and a rare missense *N1* allele (2). In contrast, the other RBPJ variants, which impact DNA binding at least 6-fold, have not been associated with other Notch pathway alleles, and to our knowledge all patients with these alleles have AOS phenotypes.

Through conditional genetics, we generated a tractable experimental model ideally suited to identify the defective N1 signaling tissues that contribute to pathogenesis. Our approach takes advantage of the fact that only mice heterozygous for both an *N1* and *Rbpj^E89G^* allele suffer pathological phenotypes. Using Cre recombination, we developed conditional mouse models that either selectively remove the *Rbpj^E89G,fl^* allele in an otherwise *N1^+/null^* background or selectively induce *N1^+/null^ Rbpj^+/E89G^* compound heterozygous genotypes in a desired tissue (Figure 5A and Figure 7A). Importantly, *Tie2-Cre*, which is expressed in endothelial and endocardial cells, rescues lethality and cardiovascular defects by deleting the *Rbpj^E89G,fl^* allele in an *N1* heterozygous background and causes lethality and cardiovascular defects by inducing *N1* heterozygosity in the presence of an *Rbpj^E89G^* allele. While these findings do not preclude the possibility that other cell types contribute to these defects, the fact that having the *N1^+/null^ Rbpj^+/E89G^* genotype in the endothelium is both necessary and sufficient to cause AOS-like phenotypes strongly suggests that defective N1-signaling in the vascular endothelium is a major driver of pathogenesis.

These findings raise new questions about what specific cellular processes during vascular and cardiac development are compromised by the RBPJ^E89G^ variant. The paucity of large yolk sac vessels in *N1^+/null^ Rbpj^+/E89G,fl^* mice suggests a failure to properly remodel the primitive vascular plexus to a hierarchically organized vascular network, a known N1-dependent process (41). In addition, the increase in hemorrhages in these embryos suggests vascular integrity is compromised, similar to that seen with anti-DLL4 antibodies (47) or N1 loss-of-heterozygosity models (48). In contrast, we did not observe obvious defects in sprouting angiogenesis as revealed by tip/stalk cell specification and vascularized branching within skin preparations. However, additional quantitative studies with temporal control using inducible Cre lines are needed to provide a better assessment of how the *Rbpj^E89G^* allele affects sprouting angiogenesis in an experimentally tractable tissue like the postnatal retina.

Similar to the vasculature, patients with AOS can have a variety of cardiac pathologies, including atrial and ventricular septal defects, valve anomalies, aortic and pulmonic stenosis, coarctation of the aorta, and tetralogy of Fallot (2). Consistent with these findings, *N1^+/null^ Rbpj^+/E89G,fl^* mice have abnormal cardiac morphology, most commonly membranous VSDs and dilated coronary vessels. The observed VSDs likely result from impaired growth or fusion of the endocardium with the cardiac neural crest–derived outflow tract septum (49). Dilated coronary vessels may be secondary to the heart failing (50) or due to aberrant patterning of vascular smooth muscle cells; the latter would be consistent with both mural cell patterning defects in patients with AOS (27) and the known role of Notch signaling in mural cell patterning (51–54). The lack of abnormal valve morphology in our mouse model is not surprising given that in mice, it is associated with modifiers such as diet (55, 56), which was not attempted in this study.

Although our study focused on defining the pathogenesis of cardiovascular defects, we were unable to similarly use our mouse model to assess the mechanisms underlying skin/scalp and limb defects, two widely regarded hallmarks of AOS in humans. In fact, throughout our mouse studies, we did not observe any obvious limb defects. However, scalp lesions were observed with one of the *N1* alleles (*N1^gKO^*) that had considerable C57/BL6 in its genetic background, raising the possibility that this phenotype is sensitive to genetic background. Thus, comparative studies are needed using inbred mice carrying conditional *N1* and *Rbpj^E89G^* alleles to isolate the role of genetic background and test whether scalp lesions are due to defective N1 signaling in endothelial and/or other cell types.

Lastly, an unanswered question is how variants in *RBPJ*, which is the sole transcription factor downstream of all NOTCH receptors, cause an N1/DLL4 syndrome (AOS) but not an N2/JAG1 syndrome (Alagille syndrome) (25). Molecularly, RBPJ is thought to similarly interact with both NICD1 and NICD2, suggesting the RBPJ AOS variants should affect both N1- and N2-dependent processes. However, we found that the *Rbpj^E89G^* allele in mice genetically interacts with *N1* alleles to cause lethality and cardiovascular defects, whereas *Rbpj^E89G^* and an *N2*-null allele were well tolerated in mice. Although additional studies are needed to assess whether *Rbpj^E89G^* can affect some N2-sensitive cell types, these data suggest that the clinical importance of the *Rbpj^E89G^* allele is due to its ability to preferentially compromise N1-dependent processes. Interestingly, comparative Notch signaling assays in cell culture revealed that ligand interactions with N2 generally produce more NICD molecules than N1 (19, 57). These studies suggest that the ratio of NICD coactivator to RBPJ transcription factor may contribute to the differential sensitivities of N1- versus N2-dependent processes to *Rbpj* AOS alleles. Importantly, the conditional mouse models generated in this study are ideally suited to assess how *Rbpj* AOS alleles affect N1- and N2-dependent processes during animal development.

## Methods

### Sex as a biological variable.

AOS occurs in males and females without obvious bias (2, 5, 6). Nevertheless, we examined male and female mice and observed similar changes in viability in both sexes (see Supporting Data Values file for the sex of mice included in postnatal viability assays). Hence, we did not consider sex as a biological variable.

### Structural modeling.

The PyMOL Molecular Graphics System (version 3.0 Schrödinger, LLC) was used to visualize the structure of RBPJ bound to DNA (Protein Data Bank assembly 3BRG) (36). We used the PyMOL mutagenesis wizard to visualize the impact of AOS-associated mutations, selecting the rotamer for each variant that occurs most frequently in proteins. Discs represent pairwise overlap of atomic van der Waals radii. The color and size of each disc correlate with the amount of overlap. All human residue numbers correspond to the numbering used in isoform Q06330-1.

### Protein purification.

A pGEX-6P-1 plasmid encoding the conserved *Rbpj* core mouse residues 53–474 was used to generate each AOS variant through QuikChange mutagenesis using the primers in Supplemental Table 3. DNA constructs were confirmed by Sanger sequencing, and proteins were purified as previously described (34, 58). Protein concentrations were determined by measuring absorbance at 280 nm using a NanoDrop spectrophotometer. Protein purity was confirmed by SDS-PAGE with GelCode Blue staining (see Supplemental Figure 1B) per the manufacturer’s protocol (Thermo Fisher Scientific, 24590).

### ITC.

ITC experiments were performed as previously described (34). Briefly, purified RBPJ proteins were assessed for binding to the following: (a) an oligonucleotide sequence 5′–GGCACCGTGGGAAACTAGTG–3′ encoding a high-affinity RBPJ site (underlined); (b) a human NOTCH1 peptide consisting of residues 1754–1781; or (c) human SHARP residues 2776–2833. The NOTCH1 peptide was synthesized as previously described (34), and human SHARP residues were cloned into pSMT3 to produce protein with an N-terminal SMT3 and His tag as previously described (59). All proteins and DNA were dialyzed overnight in a buffer containing 50 mM sodium phosphate (pH 6.5) and 150 mM sodium chloride. Experiments were done in triplicate using a MicroCal VP-ITC. RBPJ plus DNA experiments were conducted at 10°C; RBPJ plus NICD/SHARP experiments were conducted at 25°C. Experiments were performed using 20 injections of 14 μL each. Heat-of-dilution experiments were conducted by injecting each ligand (DNA, NICD, or SHARP) in the syringe into a buffer-only solution in the cell. The heat-of-dilution experiment was subtracted from the experimental data before fitting. The raw data were analyzed using ORIGIN software and fit to a 1-site binding model. A 2-tailed *t* test was used to compare WT RBPJ with each variant; a *P* value less than 0.05 indicated a significant difference.

### EMSAs.

EMSAs were performed as described previously (16, 34, 60, 61). In brief, the 5′–CGAACGAGGCAAACCTAGGCTAGAGGCACCGTGGGAAACTAGTGCGGGCGTGGCT–3′ oligonucleotide containing an RBPJ site (underlined) was annealed to a complementary 5′IRDye-700 oligonucleotide 5′–AGCCACGCCCGCACT–3′. The duplex DNA was filled in using DNA polymerase I. Binding reactions were incubated for 20 minutes at room temperature, and protein-DNA complexes were separated by acrylamide gel electrophoresis. Gels were run for 2 hours at 150 V and imaged using a LI-COR Odyssey CLx scanner. Band intensity was quantified using Image Studio software (LI-COR Biotech LLC). Each experiment was performed in triplicate. A 1-way ANOVA with Tukey’s post hoc correction was used to compare WT RBPJ with each variant; a *P* value less than 0.05 indicated a significant difference.

### Mice.

Mice carrying *Rbpj^S358R^*, *Rbpj^E89G^*, and *Rbpj^E89G,fl^* alleles were made in collaboration with the Cincinnati Children’s Hospital Medical Center Transgenic Animal and Genome Editing Facility (TAGE, RRID:SCR_022642) using CRISPR/Cas9 genome editing. For the *Rbpj^S358R^* allele, we targeted cleavage to a site surrounding the S358 codon with the sgRNA 5′–TCCCTCATAGAACGTGTACTCGG–3′ and introduced a donor oligonucleotide 5′–ATCATTAGAACTGATAAAGCTGAGTATACG–3′ that substituted an arginine in place of S358 and introduced a DdeI restriction site for genotyping. For *Rbpj^E89G^* and *Rbpj^E89G,fl^*, we targeted cleavage to a site surrounding the E89 codon with the sgRNA 5′–AGTCTTACGGAAATGAAAAACGG–3′ and introduced a donor oligonucleotide 5′–CAGAAGTCATATGGGAATGGAAAA–3′ that substituted a glycine in place of E89 and introduced an NdeI restriction site for genotyping. *Rbpj^E89G^* was made by editing WT CD1 mice; *Rbpj^E89G,fl^* was made in outbred mice with existing flox sites surrounding exons 6 and 7 of the *Rbpj* gene (37). The genotypes of founder animals were confirmed using Sanger sequencing.

The other mouse lines used in this study included 3 *N1* alleles: *N1^tm1Con^* (38) deletes genomic regions encoding amino acids 1056–2049, which includes the entire transmembrane region and Ankyrin repeats, and therefore is considered a constitutive null allele (*N1^null^*). The *N1^tm2Agt^* allele (39) was generated by incorporating loxP sites flanking the promoter and part of exon 1 followed by Cre recombination in the germline to make a constitutive *N1*-null allele referred to as *N1^gKO^*. The *N1^tm2Rko^* allele (44) was independently made in-house by inserting loxP sites in nearly identical sequences as Radtke et al. (39). We refer to this conditional allele as *N1^fl/fl^*. The other alleles used in this study were *Rbpj^null^* (62), *Rbpj^fl/fl^* (37), *N2^LacZ^* (63), and *Tie2-Cre^Ywa^* (42). Offspring were genotyped using primers listed in Supplemental Table 2.

### Timed embryonic harvest.

Gestation was timed such that observation of a vaginal plug was considered E0.5. Pregnant dams were euthanized via CO_2_ inhalation followed by cervical dislocation, and the uterus was removed and placed into PBS on ice. Embryos were harvested and imaged with a Nikon SMZ 1500 stereoscope prior to collection of tissues. Specifically, the forelimbs, head, heart, and/or yolk sac were collected for analysis and placed into 4% paraformaldehyde (PFA) in PBS and incubated at 4°C overnight.

### Western blotting.

Single E10.5 *Rbpj^+/+^* and *Rbpj^E89G,fl/E89G,fl^* embryos were homogenized in 2x Laemmli sample buffer for Western blot analysis. Samples were sonicated and stored at –80°C. Protein extracts (whole embryos for *Rbpj^E89G,fl/E89G,fl^* homozygotes, one-quarter embryos for WT controls) were run on a Bio-Rad 4%–20% Mini-PROTEAN TGX Stain-Free Precast Gel (catalog 456-8093) and transferred to a PVDF membrane via semidry transfer. The membrane was washed with water and then PBS before blocking with 0.5% casein in PBS for 1 hour at room temperature. The membrane was subsequently washed in PBS with 0.1% Tween-20, blocked in 0.5% casein with 0.05% Tween-20 in PBS (pH 7.4) for 1 hour at room temperature, and then incubated with antibodies against RBPJ (1:1,000, Cell Signaling Technology, 5313) and β-actin (1:2,000, LI-COR, 926-42212) overnight at 4°C. The membrane was washed in PBS with 0.1% Tween-20 and incubated with secondary antibodies (1:4,000 goat anti-rabbit IgG AF555, Invitrogen, A-21429; and 1:4,000 donkey anti-mouse IgG 680RD, LI-COR, 926-68072) at room temperature for 90 minutes. Finally, the membrane was washed in PBS with 0.1% Tween-20 and imaged using a Bio-Rad ChemiDoc imaging system. Band intensity was quantified using the Image Lab Software Suite (Bio-Rad), and RBPJ was normalized to β-actin levels.

### Embryonic and postnatal heart assays.

After overnight fixation in 4% PFA, E16.5 or postnatal hearts were washed 3 times for 5 minutes in PBS and submitted to the Integrated Pathology Research Facility for processing and embedding in paraffin (RRID:SCR_022637). Hearts were serially sectioned and either stained with H&E as described previously (64) or blocked and stained with 1:100 VE-cadherin (R&D Systems, AF1002). Stained heart sections were imaged using a Nikon NiE upright widefield microscope or Nikon A1R inverted confocal microscope.

### Yolk sac vascular assays.

E14.5 or E16.5 embryos were harvested and imaged within their yolk sacs from multiple angles with a Nikon SMZ 1500 stereoscope. Yolk sac vasculature was considered “reduced” if vitelline vessels were absent or markedly narrowed and/or if the visible capillary plexus extended over less than half of the yolk sac surface area. Yolk sac vasculature was scored by researchers blinded to genotype.

E10.5 embryos were fixed within their yolk sacs in 4% PFA in PBS for 30–60 minutes at room temperature. Embryos were washed 3 times for 5 minutes in PBS, dissected out of their yolk sacs, and reserved for genotyping. Empty yolk sacs were fixed in 4% PFA in PBS overnight at 4°C, washed 3 times for 5 minutes in PBT (PBS + 0.2% Triton X-100), blocked with 10% donkey serum in PBT for 2 hours at room temperature, and incubated with a rat anti-CD31 antibody (1:300, BD Biosciences, 553369) for 3 days at 4°C. Yolk sacs were washed 5 times for 15 minutes at room temperature with 2% normal donkey serum in PBT and incubated with a secondary antibody (1:300 donkey anti-rat AF647, Jackson ImmunoResearch Laboratories Inc., 712-605-153) for 2 days at 4°C. Yolk sacs were again washed 5 times for 15 minutes at room temperature and float-mounted in 1% agarose in coverslip-bottomed 48-well plates (Mattek, P48G-1.5-6). Tissue clearing was performed by adding 200 μL of EZClear (65) and incubating overnight prior to imaging with a Nikon A1R inverted confocal microscope. Image analysis and quantification were performed with AngioTool software (66). For calculating the percentage of vascular coverage, binaries were created for CD31-stained areas, and the relative coverage of the binaries compared with total image area was determined. For vascular diameter distributions, representative 400 μM × 400 μM areas were chosen and vessel diameters between all branch points were measured using the NIS-Elements measurements tool .

### Embryonic skin vascular assays.

Embryonic skin assays were performed essentially as previously described (67). In brief, PFA was removed from E14.5 forelimbs and heads by washing 3 times for 5 minutes in PBS. Tissues were transferred to 100% methanol (MeOH) for storage at –20°C. Using forceps, the skin was removed from the forelimbs and heads and rehydrated through a graded series of MeOH/PBT (PBS + 0.2% Triton X-100) washes. Skins were blocked with 10% donkey serum in PBT for 2 hours at room temperature and incubated with a rat anti-CD31 antibody (1:300, BD Biosciences, 553369) overnight at 4°C. Skins were then washed 5 times for 15 minutes at room temperature with 2% donkey serum in PBT and incubated with a secondary antibody (1:300 donkey anti-rat AF647, Jackson ImmunoResearch Laboratories Inc.,712-605-153) for 1 hour at room temperature. Skins were washed 5 times for 15 minutes at room temperature, mounted on slides, and imaged using a Nikon A1R inverted confocal microscope. Image analysis and quantification were performed with AngioTool (66) and Imaris software.

### Statistics.

Mouse viability was analyzed using the χ^2^ test for deviation from expected Mendelian ratios. Fisher’s exact test was used to determine whether the frequency of a phenotype differed between groups. Additional statistical tests are described in corresponding figure legends. For all statistical tests, a *P* value less than 0.05 indicated a significant difference.

### Study approval.

Animal experiments were carried out under protocols approved by the IACUC at Cincinnati Children’s Hospital Medical Center (protocols 2016-0105 and 2021-0086).

### Data availability.

All values underlying graphed data are available in the Supporting Data Values file.

## Author contributions

RAK, RK, and BG conceptualized the study. AFS, KP, BC, HWL, CA, and EKG conducted formal analysis. BG acquired funding. AFS, KP, BC, RH, PG, ZY, BB, GM, HN, and EKG conducted the investigation. AFS, KP, BC, ZY, and EKG devised the methodology. RAK, RK, and BG were responsible for project administration. ZY, LS, and RAK provided resources. EKG, RAK, RK, and BG supervised the study. AFS and BG prepared the original draft. AFS, KP, BC, EKG, LS, RK, and BG reviewed and edited the manuscript.

## Funding support

This work is the result of NIH funding, in whole or in part, and is subject to the NIH Public Access Policy. Through acceptance of this federal funding, the NIH has been given a right to make the work publicly available in PubMed Central.

- NIH (R01GM079428 to BG).
- National Science Foundation grant (2114950 to BG).

## Supplementary Material

Supplemental data

Unedited blot and gel images

Supporting data values

## Figures and Tables

**Table 1:**
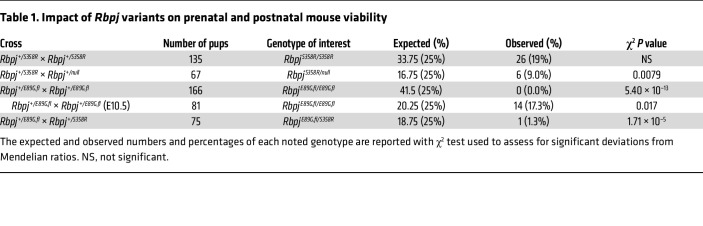
Impact of *Rbpj* variants on prenatal and postnatal mouse viability

**Table 2:**
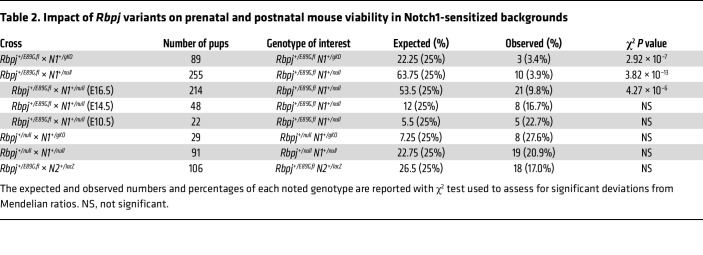
Impact of *Rbpj* variants on prenatal and postnatal mouse viability in Notch1-sensitized backgrounds

**Table 3:**
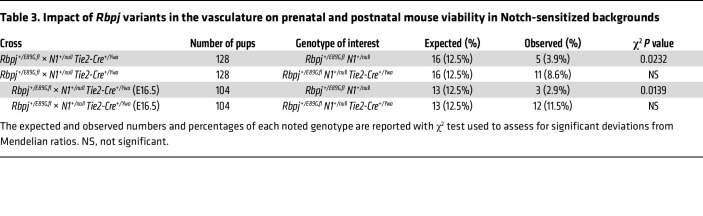
Impact of *Rbpj* variants in the vasculature on prenatal and postnatal mouse viability in Notch-sensitized backgrounds

**Table 4:**
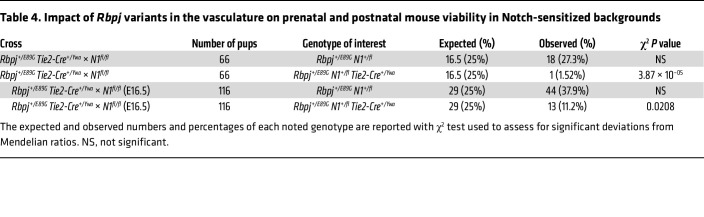
Impact of *Rbpj* variants in the vasculature on prenatal and postnatal mouse viability in Notch-sensitized backgrounds

## Version 1 10/07/2025

In-Press Preview

## Version 2 12/01/2025

Electronic publication
