# Supplementary material for: Defective Notch1 signaling in endothelial cells drives pathogenesis in a mouse model of Adams-Oliver syndrome
Source: J Clin Invest. 2025 Oct 7;135(23):e187532. doi: 10.1172/JCI187532 (PMC12646667; doi:10.1172/JCI187532)
Supplement: Supplemental data [file jci-135-187532-s095.pdf]

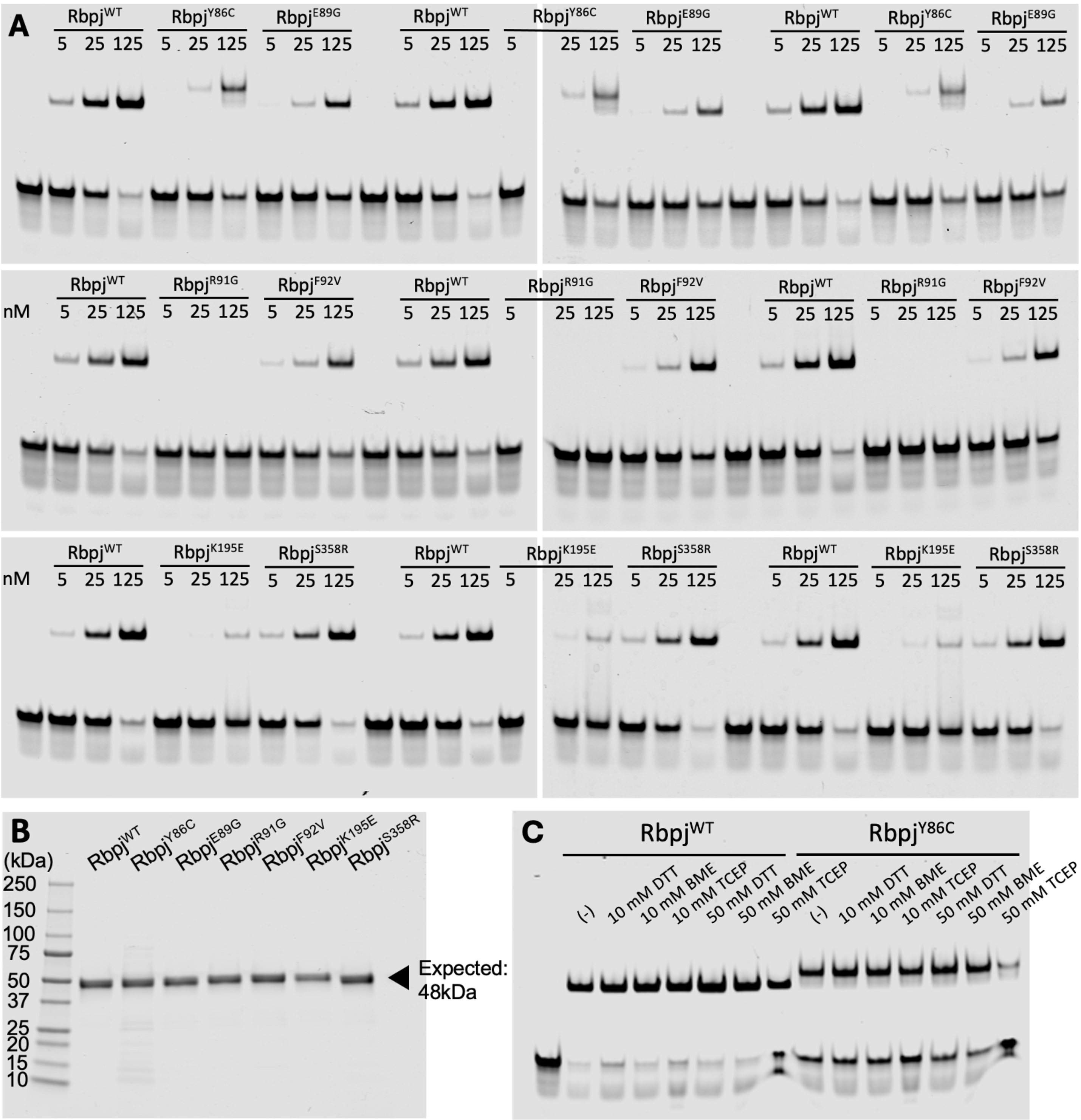

Figure S1: DNA binding analysis of AOS-associated RBPJ variants using EMSAs. A) Triplicate EMSAs of wild-type RBPJ and RBPJ variants Y86C, E89G, R91G, F92V, K195E, and S358R. The DNA probe contained a single high affinity RBPJ binding site (CGTGGGAA). Quantitative statistical analysis of probe depletion is presented in Figure 1I. B) SDS-PAGE with GelCode Blue staining of wild-type and variant RBPJ protein preparations shows a single 48 kDa band in each lane. C) EMSA of wild-type Rbpj and Rbpj variant Y86C (125 nM) with and without the indicated concentrations of reducing agents dithiothreitol (DTT), beta-mercaptoethanol (BME), and tris(2-carboxyethyl)phosphine (TCEP).

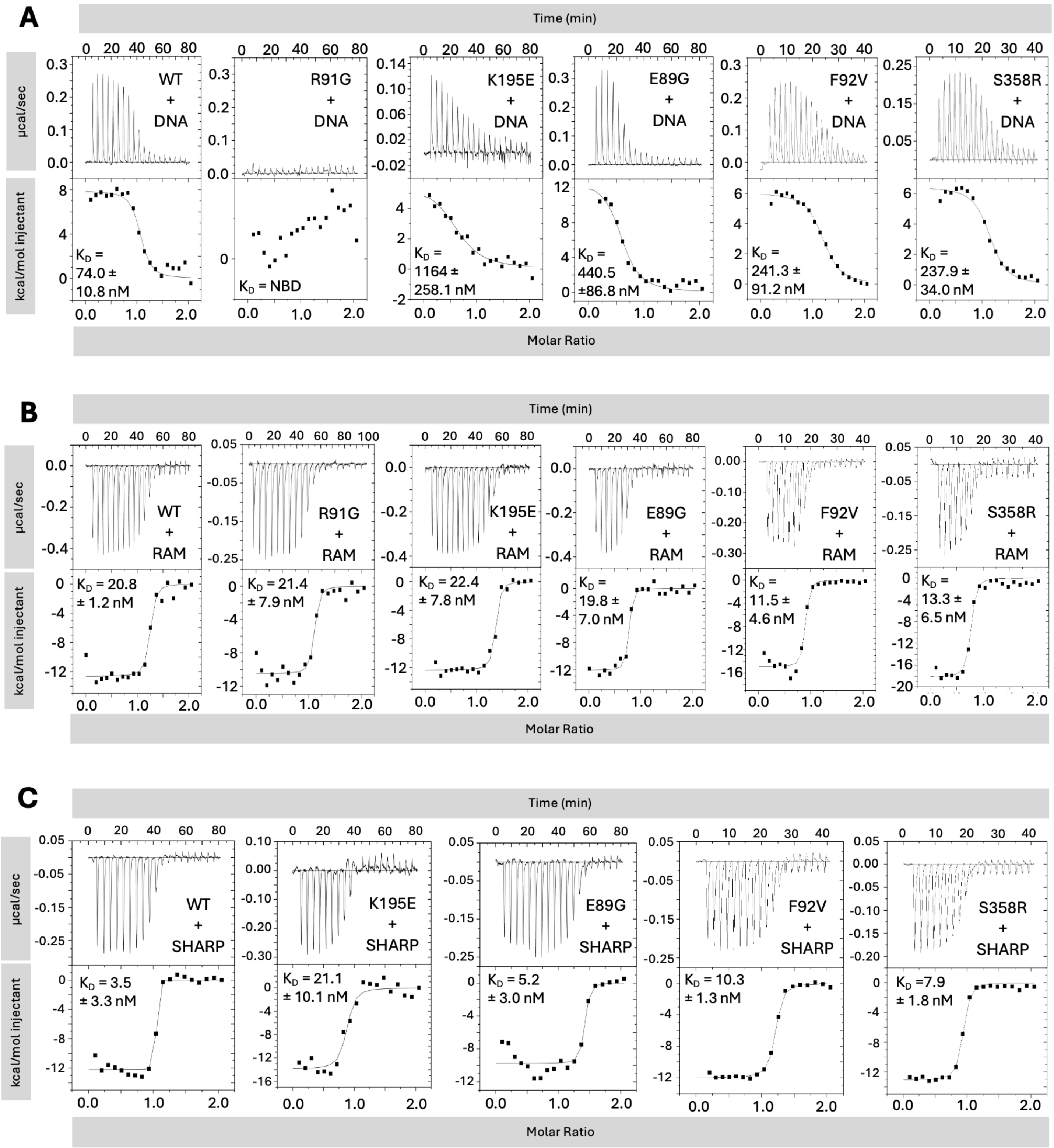
A
Time (min)
µcal/sec
kcal/mol injectant
Molar Ratio
WT + DNA
K_D = 74.0 ± 10.8 nM
R91G + DNA
K_D = NBD
K195E + DNA
K_D = 1164 ± 258.1 nM
E89G + DNA
K_D = 440.5 ±86.8 nM
F92V + DNA
K_D = 241.3 ± 91.2 nM
S358R + DNA
K_D = 237.9 ± 34.0 nM
B
Time (min)
µcal/sec
kcal/mol injectant
Molar Ratio
WT + RAM
K_D = 20.8 ± 1.2 nM
R91G + RAM
K_D = 21.4 ± 7.9 nM
K195E + RAM
K_D = 22.4 ± 7.8 nM
E89G + RAM
K_D = 19.8 ± 7.0 nM
F92V + RAM
K_D = 11.5 ± 4.6 nM
S358R + RAM
K_D = 13.3 ± 6.5 nM
C
Time (min)
µcal/sec
kcal/mol injectant
Molar Ratio
WT + SHARP
K_D = 3.5 ± 3.3 nM
K195E + SHARP
K_D = 21.1 ± 10.1 nM
E89G + SHARP
K_D = 5.2 ± 3.0 nM
F92V + SHARP
K_D = 10.3 ± 1.3 nM
S358R + SHARP
K_D =7.9 ± 1.8 nM

Figure S2: Isothermal titration calorimetry studies show that AOS-associated RBPJ variants impair binding to DNA but not to co-factors. Representative ITC thermograms showing the raw heat signal and nonlinear least squares fit to the data for mouse RBPJ variants binding to: A) an oligonucleotide containing a high-affinity RBPJ-binding site; B) the RBPJ association module (RAM) domain (VLLSRKRRRQHGQLWFPEGFKVSEASKK) of the human Notch1 intracellular domain; and C) a fusion protein of SMT3-SHARP 2776-2833 corresponding to the region of the human SHARP co-repressor protein that binds RBPJ. For experiments with wild-type (WT) RBPJ and the R91G, K195E, and E89G variants, the ligands (DNA, RAM, or SHARP) were placed in the syringe at 100μM and RBPJ protein was placed in the cell at 10μM. For experiments with the F92V variant, the ligand was placed in the syringe at 50μM and the Rbpj protein was placed in the cell at 5μM. For experiments with the S358R variant, the ligand was placed in the syringe at 40μM and the Rbpj protein was placed in the cell at 4μM. The mean dissociation constants (K_D) and standard deviations from triplicate experiments are reported. NBD = no binding detected. Statistical analysis of the DNA binding data is presented in Figure 1J and the statistical analysis of cofactor binding is presented in Table S1.

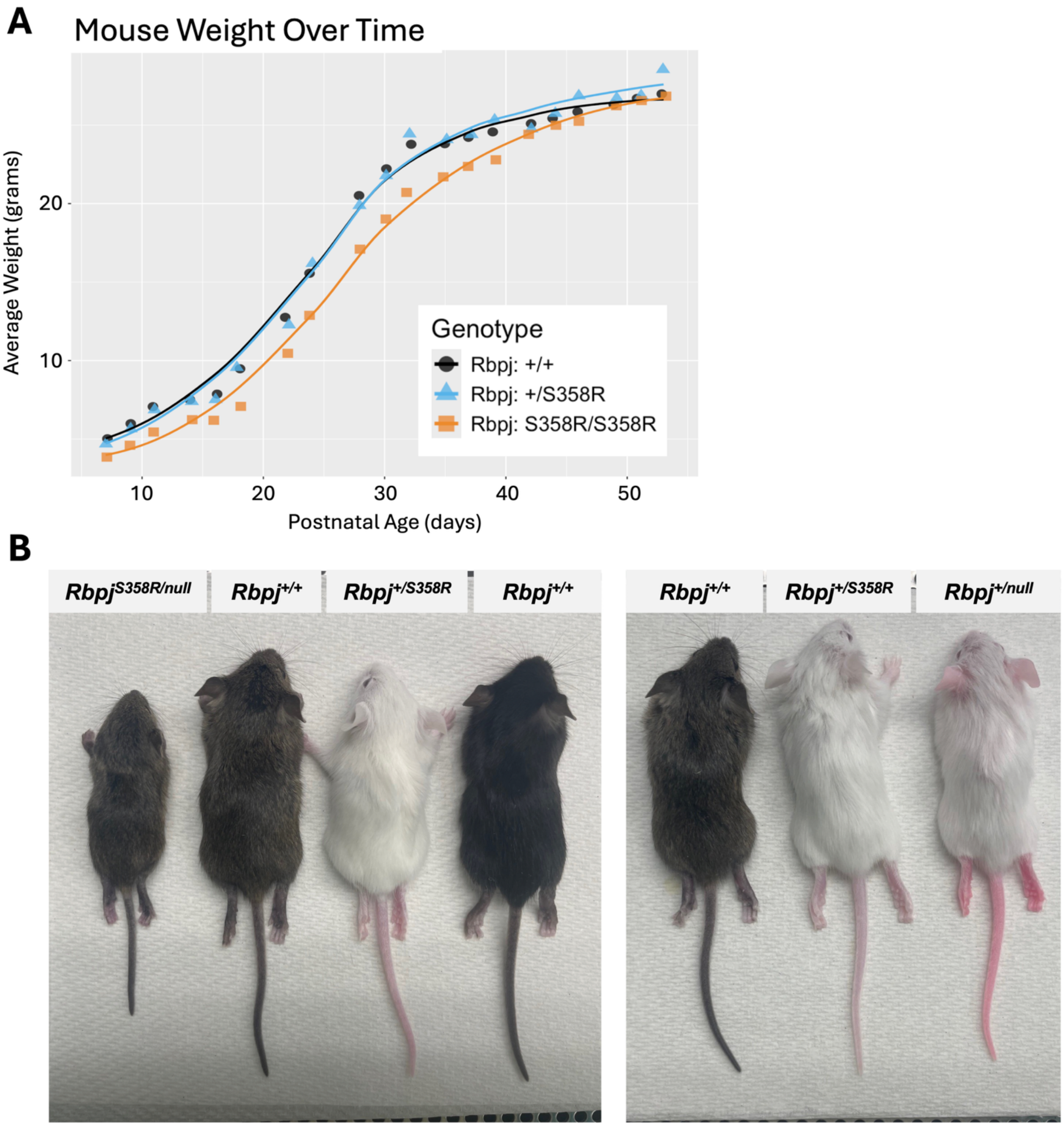

Figure S3: $Rbpj^{S358R}$ is a mild hypomorphic allele in mice. A) Plot of average body weight over time for $Rbpj^{+/+}$ (n = 13), $Rbpj^{+/S358R}$ (n = 25), and $Rbpj^{S358R/S358R}$ (n = 10) mice showing significant growth delay in $Rbpj^{S358R/S358R}$, but not $Rbpj^{+/S358R}$. Statistical analysis was performed using a two-way mixed ANOVA, which revealed that the interaction between genotype and postnatal age had a significant impact on weight (p = 2.09e-05). To further explore this interaction, we performed one-way ANOVAs at each timepoint with a Bonferroni correction for multiple comparisons and found weights were significantly different across genotypes until postnatal day 28. Pairwise comparisons from a Tukey post-hoc revealed that $Rbpj^{+/+}$ and $Rbpj^{S358R/S358R}$ were significantly different at these timepoints. B) $Rbpj^{S358R/null}$ hemizygotes are smaller than their $Rbpj^{+/+}$, $Rbpj^{+/S358R}$, and $Rbpj^{+/null}$ littermates.

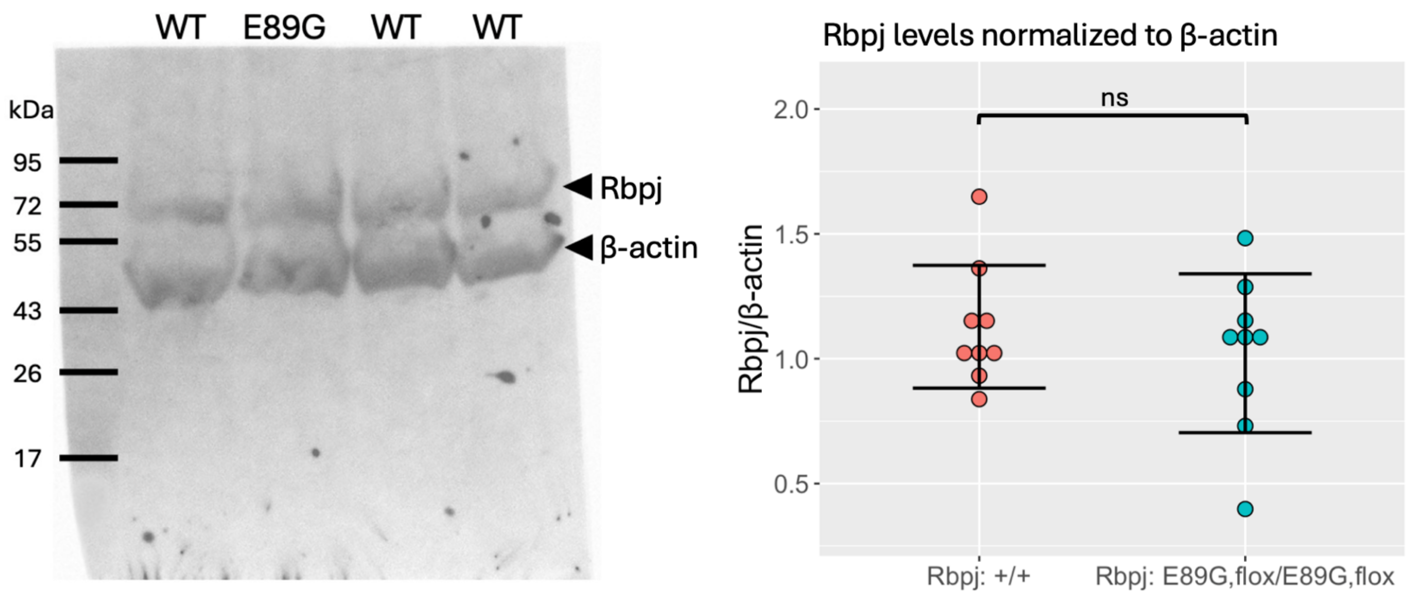

Figure S4: $RBPJ^{E89G}$ is expressed at wild-type RBPJ levels. (*Left*) Representative western blot of protein extracts from *Rbpj*$^{+/+}$ (n = 9) and *Rbpj*$^{E89G,flox/E89G,flox}$ (n = 9) embryos at E10.5 stained with antibodies against RBPJ and ACTB (beta-actin). (*Right*) Plot with quantification of RBPJ levels normalized to ACTB. Error bars encompass the standard deviation from the mean. A two-tailed t-test revealed no significant difference in RBPJ levels between genotypes.

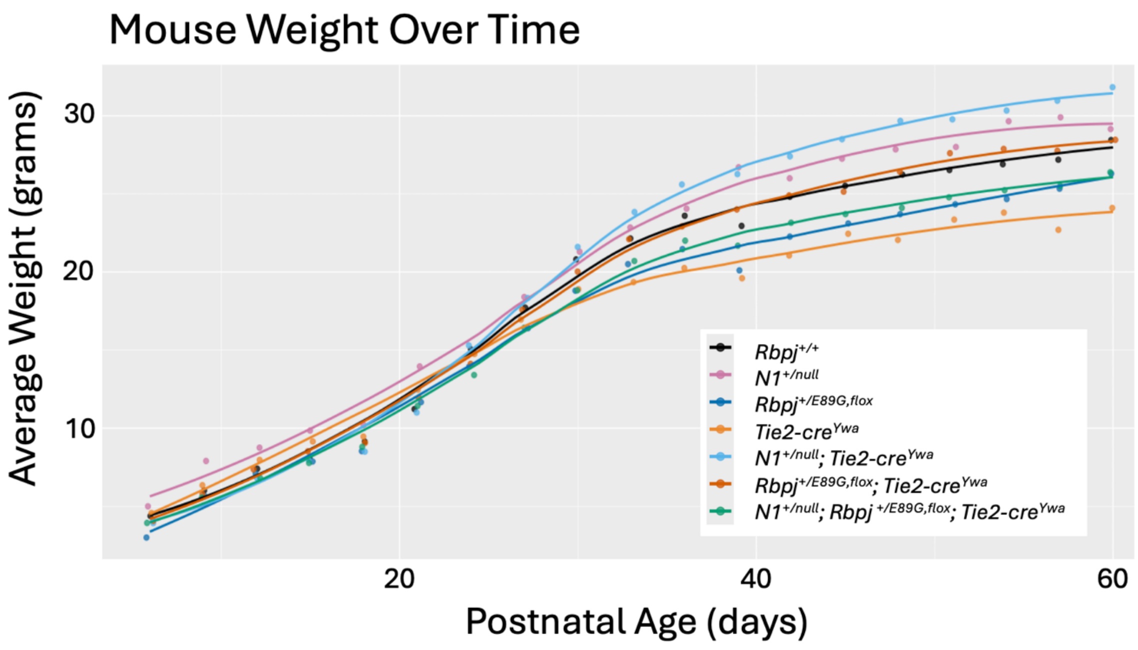

Figure S5: *N1*$^{+/null}$*;Rbpj*$^{+E89G,flox}$*;Tie2-cre*^{Ywa} mice do not show postnatal growth delay. Wild-type (n = 7), *N1*$^{+/null}$ (n = 3), *Rbpj*$^{+/E89G,flox}$ (n = 5), *Tie2-cre*^{Ywa} (n = 3), *N1*$^{+/null}$*;Tie2-cre*^{Ywa} (n = 5), *Rbpj*$^{+/E89G,flox}$*;Tie2-cre*^{Ywa} (n = 7), and *N1*$^{+/null}$*;Rbpj*$^{+/E89G,flox}$*;Tie2-cre*^{Ywa} (n = 9) mice were weighed every 3 days from postnatal day 6 (P6) through postnatal day 60 (P60). *N1*$^{+/null}$*;Rbpj*$^{+/E89G,flox}$ mice could not be assessed due to insufficient numbers surviving postnatally. Two-way mixed ANOVA revealed that neither genotype nor the interaction between genotype and postnatal age had a statistically significant effect on weight.

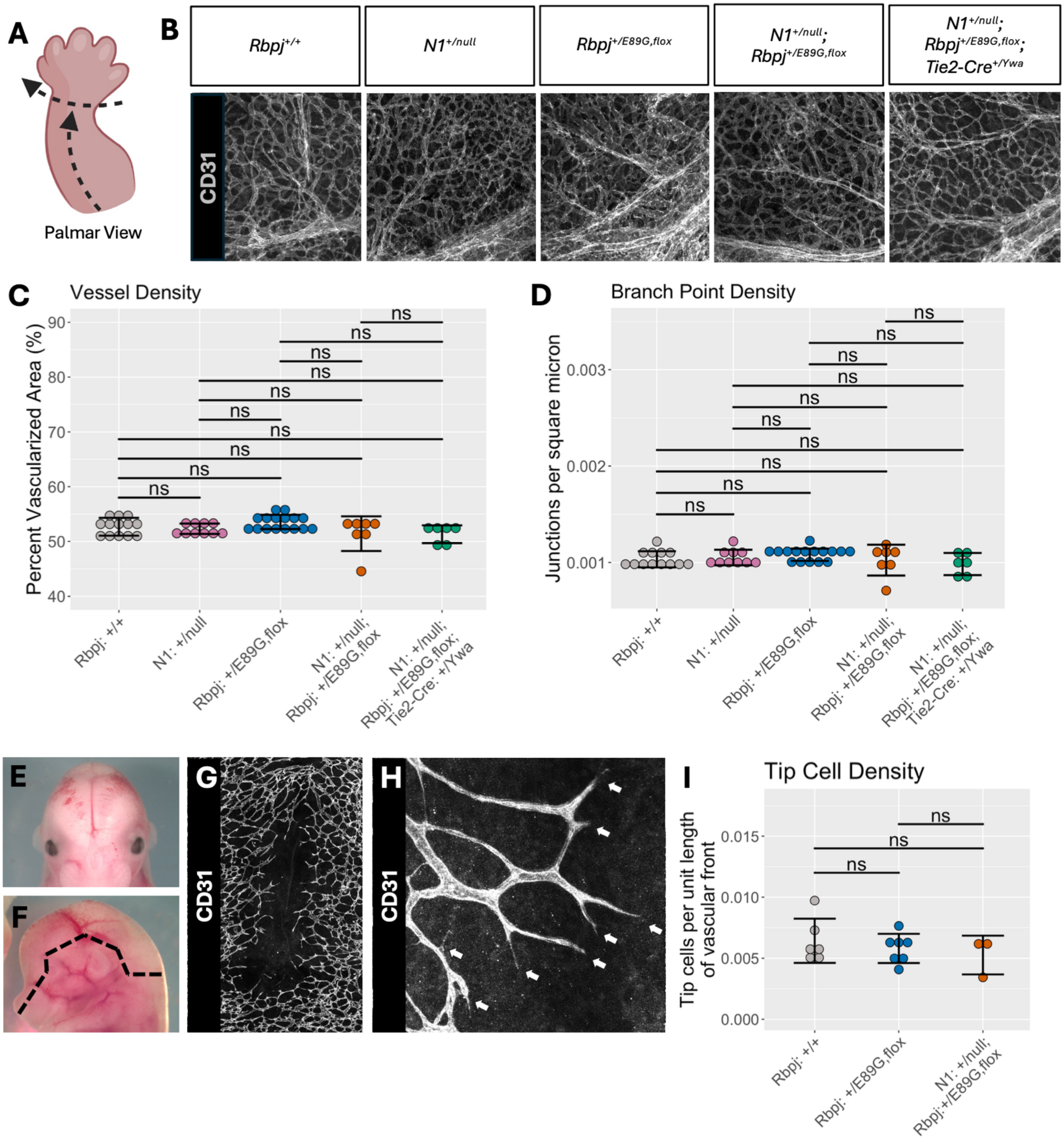

A
Palmar View
B
Rbpj+/+
N1+/null
Rbpj+/E89G,flox
N1+/null; Rbpj+/E89G,flox
N1+/null; Rbpj+/E89G,flox; Tie2-Cre+/Ywa
CD31
C
Vessel Density
Percent Vascularized Area (%)
ns
Rbpj: +/+
N1: +/null
Rbpj: +/E89G,flox
N1: +/null; Rbpj: +/E89G,flox
N1: +/null; Rbpj: +/E89G,flox; Tie2-Cre: +/Ywa
D
Branch Point Density
Junctions per square micron
E
F
G
CD31
H
CD31
I
Tip Cell Density
Tip cells per unit length of vascular front
Rbpj: +/+
Rbpj: +/E89G,flox
N1: +/null; Rbpj:+/E89G,flox

Figure S6: Skin vascular assays show no difference between *N1*$^{+/null}$*; Rbpj*$^{+/E89G,flox}$ embryos and littermates. A) Schematic for removal of skin from forelimbs of E14.5 embryos by cutting from proximal to distal down the ventral side of the forelimb and circumferentially around the wrist. *Created in BioRender. Solano, A. (2025) https://BioRender.com/em90lrj*. B) Representative images of E14.5 forelimb skin vasculature stained by CD31 at 20X magnification. C-D) Graphs showing the quantification of vessel density (C) and branch point density (D) in E14.5 forelimb skin vasculature. Groups were compared using two-way ANOVA followed by two-tailed t-tests with Bonferroni correction. E) Representative image of an E14.5 embryo with active vascular extension towards the midline scalp. F) Schematic for removal of scalps from E14.5 embryos by cutting along dashed lines on both sides of the head. G) Representative image of an E14.5 scalp with vessels stained with CD31 at 20X magnification. Note midline unvascularized area and active vascular extension towards the midline. H) Tip cells (white arrows) at the vascular front are identifiable by long, thin extensions. I) Graph showing the quantification of tip cell density at the scalp midline vascular front in E14.5 embryos. Two-tailed t-tests with Bonferroni correction showed no significant difference in tip cell density between genotypes. NS = not significant. All graphs display individual animals and error bars show Standard deviation from the mean.

Rbpj	Ligand	K_D (nM)	Fold Change	P-value	Study
$RBPJ^{WT}$	NICD	20.8 ± 1.2	N/A	N/A	Gagliani et al., 2022
$RBPJ^{S358R}$		13.3 ± 6.5	~0.6	NS	This Study
$RBPJ^{F92V}$		11.5 ± 4.6	~0.6	NS	This Study
$RBPJ^{E89G}$		19.8 ± 7.0	~1	NS	Gagliani et al., 2022
$RBPJ^{K195E}$		22.4 ± 7.8	~1.1	NS	Gagliani et al., 2022
$RBPJ^{R91G}$		21.4 ± 7.9	~1	NS	This Study
$RBPJ^{WT}$	SHARP	3.5 ± 3.3	N/A	N/A	Gagliani et al., 2022
$RBPJ^{S358R}$		7.9 ± 1.8	~2.3	NS	This Study
$RBPJ^{F92V}$		10.3 ± 1.3	~2.9	NS	This Study
$RBPJ^{E89G}$		5.2 ± 3.0	~1.5	NS	Gagliani et al., 2022
$RBPJ^{K195E}$		21.1 ± 10.1	~6	NS	Gagliani et al., 2022

Table S1: Isothermal titration calorimetry studies show that AOS-associated RBPJ variants do not significantly impact binding affinity to cofactors. Tabulated ITC binding data of RBPJ variants to the Rbpj association module of human NOTCH1 intracellular domain (NICD) and the corepressor SHARP. Fold change is calculated relative to wild-type Rbpj. A two-tailed t-test was used to compare the dissociation constant (K_D) of wild-type RBPJ to each variant. N/A = not applicable. NS = not significant.

Purpose	Primer Sequences
Cloning	GGAGGATCCCCACCCAAACGACTCACTAG
	GGTCTCGAGTTATGGCTCTGGTGTGTAGGTG
Introduce Y86C	AAATCGTTTTTCATTTCCGCAAGACTTCTGTGCAACTTTTGCATG
	CATGCAAAAGTTGCACAGAAGTCTTGCGGAAATGAAAAACGATTT
Introduce E89G	GGAGGGCAAAAAAATCGTTTTCCATTTCCGTAAGACTTCTGTG
	CACAGAAGTCTTACGGAAATGGAAAACGATTTTTTTGCCCTCC
Introduce R91G	GGAGGAGGGCAAAAAAATCCTTTTTCATTTCCGTAAGAC
	GTCTTACGGAAATGAAAAAGGATTTTTTTGCCCTCCTCC
Introduce F92V	CAAGGAGGAGGGCAAAAAACTCGTTTTTCATTTCCGTAAG
	CTTACGGAAATGAAAAACGAGTTTTTTGCCCTCCTCCTTG
Introduce K195E	TCTTCAGTGACTGCTTCTTCTCGGAGGGTTTGGAGATGACC
	GGTCATCTCCAAACCCTCCGAGAAGAAGCAGTCACTGAAGA
Introduce S358R	GTATTCTGCCTTGTCTGTCCTAATGATTGTCCAGGAA
	TTCCTGGACAATCATTAGGACAGACAAGGCAGAATAC

Table S2: Primers used for site-directed mutagenesis. Primer sequences are listed 5’ – 3’.

Gene Variant		Primer Sequences	Annealing Temp. (℃)	Restriction Enzyme Digest	Product Size (bp)
***Notch1**^{gKO}*					
	N1-F	CTGACTTAGTAGGGGGAAAAC	64	(none)	~400
	N1-R	AATCAGAGCGGCCCATTGTCG			
***Notch1**^{null}*					
	N1-F	ACGATATCGTGGTGCATACGCTCCTGTGCG	64	(none)	~400
	N1-R	GTCAGTTTCATAGCCTGAAGAACG			
***Rbpj**^{E89G}* **and** ***Rbpj**^{E89G,flox}*					
	Rbpj-flox-F	TGTTTGCCACCAGAATCTGTTTGTTATTTGC	68	(none)	WT: 198<br>Floxed: 248
	Rbpj-flox-R	AGGTACCTGGTACTAACTGTCTGGGACCG			
	Rbpj-E89G-F	AGCATTCCTCTCATCTGCTAGAG	62	NdeI	Uncut: 275<br>Cut (WT): 231/44<br>Cut (E89G): 164/67/44
	Rbpj-E89G-R	TGAACCAAGGAATAGAACCAAGTC			
***Rbpj**^{S358R}*					
	Rbpj-S358R-F	CTCAAGTAGGTGAATGTCACAGAG	60	DdeI	Uncut (WT): 286<br>Cut (S358R): 168/105/13
	Rbpj-S358R-R	GGCAAGTTATAGCTCAGAACAGC			
***Rbpj**^{null}*					
	Rbpj-null-F	TGGCACTGTTCAATCGCCTT	60	(none)	~350
	Rbpj-null-R	GAGGAAATTGCATCGCATTGTCTGAG			
***Tie2-Cre**^{Ywa}*					
	Tie2-Cre-F	GGGAAGTCGCAAAGTTGTGAG	59	(none)	~350
	Tie2-Cre-R	TAACCAGTGAAACAGCATTGC			

Table S3: Genotyping information for mouse lines. Primer sequences are listed 5’ – 3’. F = forward, R = reverse, WT = wild-type, bp = base pairs.
