# Supplementary material for: Defective Notch1 signaling in endothelial cells drives pathogenesis in a mouse model of Adams-Oliver syndrome
Source: J Clin Invest. 2025 Oct 7;135(23):e187532. doi: 10.1172/JCI187532 (PMC12646667; doi:10.1172/JCI187532)

Uncropped EMSA images used in Fig. 1 and Fig. S1A,
areas for Fig.1 boxed red (C-H labeled), S1A boxed blue

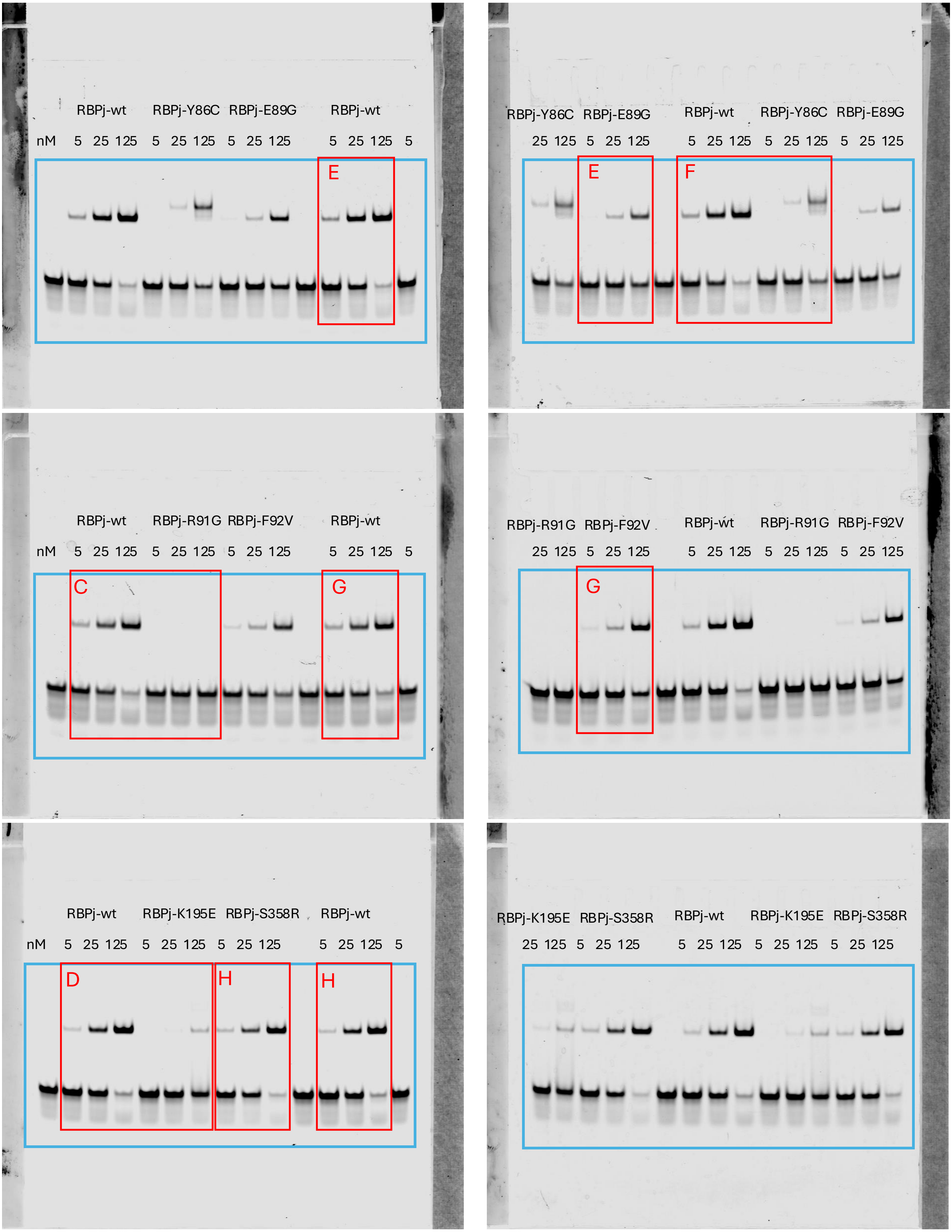

Uncropped image used in Fig. S1B, used area boxed blue

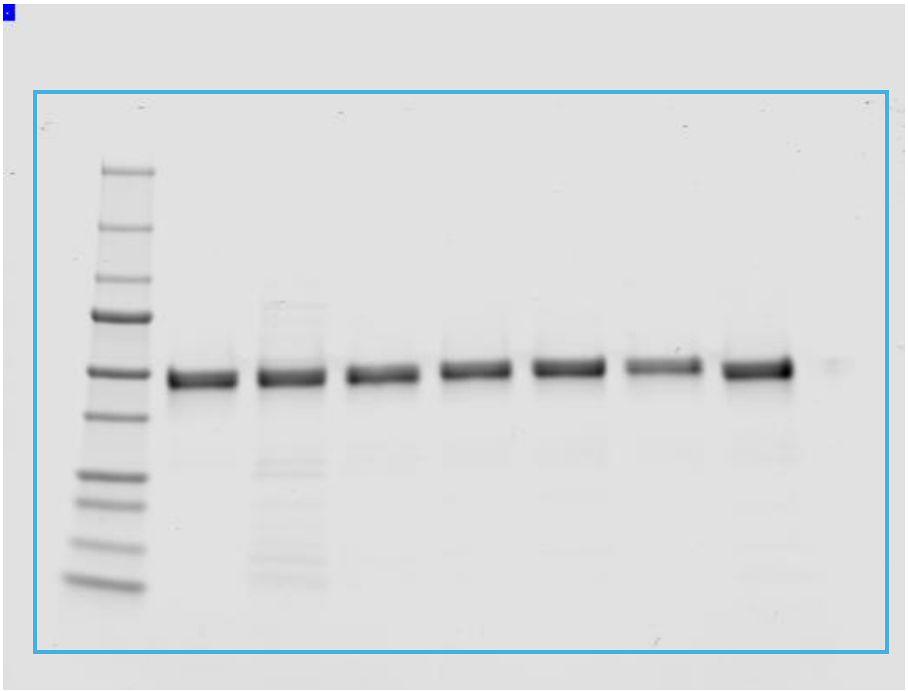

Uncropped image used in Fig. S1C, used area boxed blue

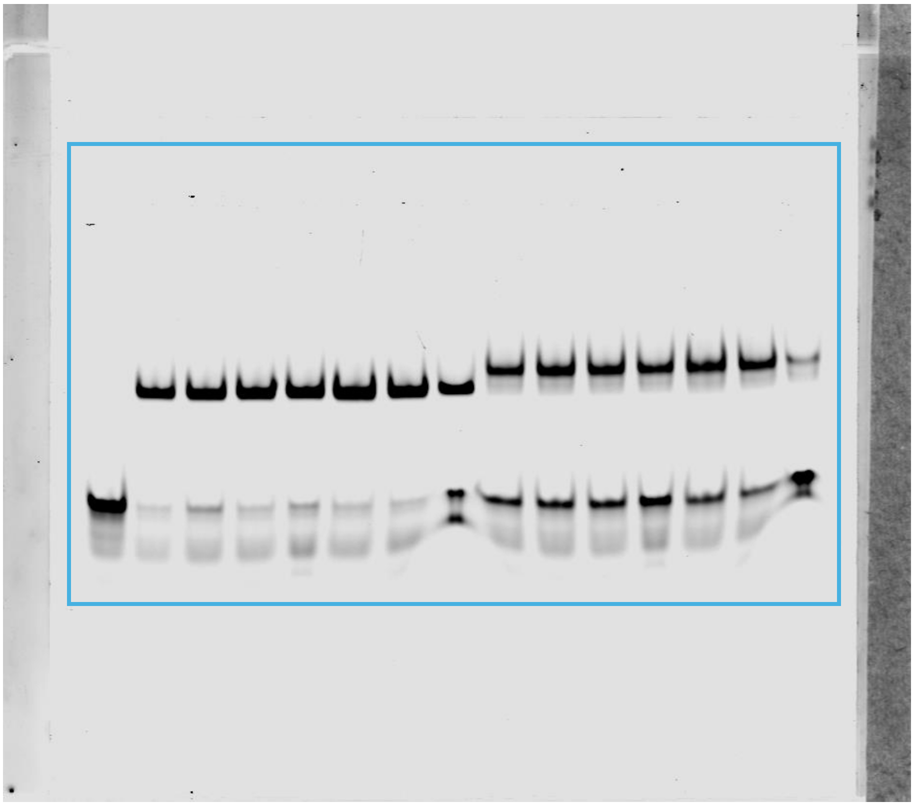

Uncropped Western blot images (Fig S4)

Gray scale, used for Fig S4

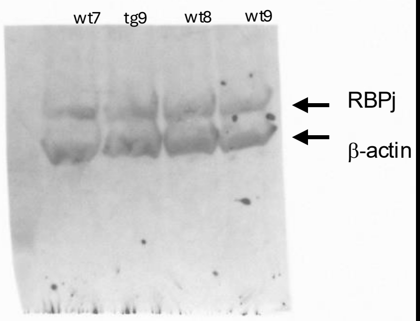

Multi color (β-actin – blue, RBPj – red)

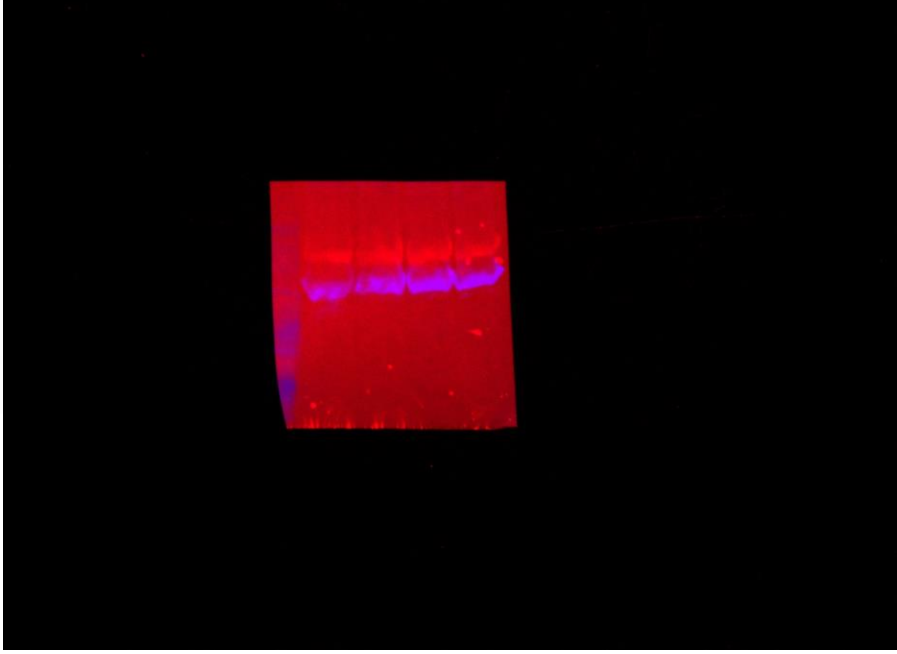

Additional Uncropped Western blot images used for quantification (Fig S4)

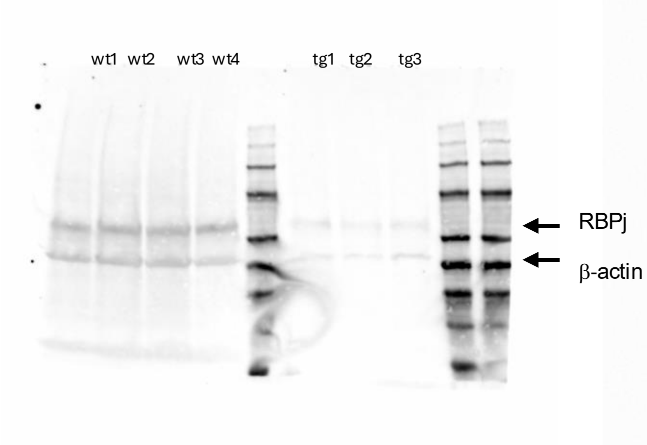

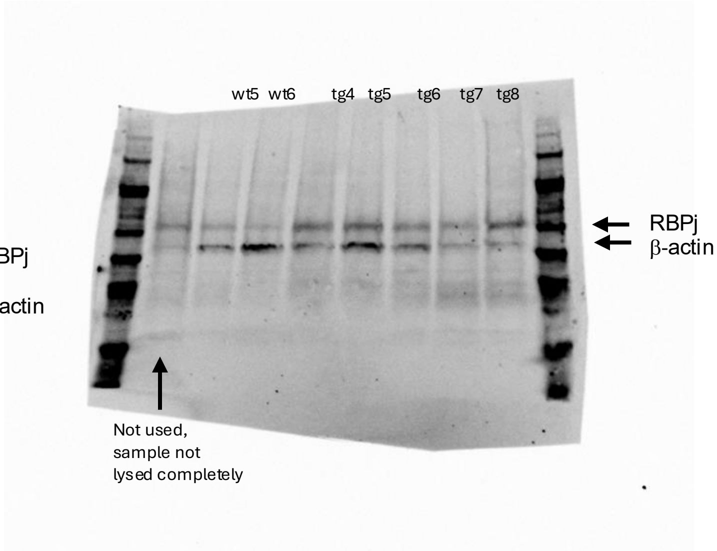

Supplement: Unedited blot and gel images [file jci-135-187532-s096.pdf]
